# Comparative diagnostic accuracy of radiomics, deep learning, and hybrid AI for invasiveness stratification of pulmonary ground-glass nodules: a systematic review and meta-analysis

**DOI:** 10.3389/fonc.2026.1752652

**Published:** 2026-07-24

**Authors:** Ning Dong, Zhongwei Li, Zhenjie Cong, Xinru Ba, Jing Qin, Zhuxiao Lin, Xiaolin Wang, Peng Liang

**Affiliations:** Department of Radiology, Yantaishan Hospital, Yantai, China

**Keywords:** deep learning, diagnostic accuracy, ground-glass nodule, hybrid artificial intelligence, lung adenocarcinoma, radiomics, systematic review

## Abstract

**Background:**

Accurate preoperative stratification of ground-glass nodules (GGNs) into invasive versus non-invasive adenocarcinomas is essential for optimizing surgical management and preventing overtreatment. Radiomics, deep learning, and hybrid models have shown potential for noninvasive histopathologic prediction, but their comparative diagnostic performance and generalizability remain unclear. This study systematically compares the diagnostic accuracy, methodological quality, and clinical applicability of radiomics, deep learning, and hybrid AI approaches for differentiating invasive from non-invasive lung adenocarcinomas manifesting as GGNs.

**Methods:**

A comprehensive search of PubMed, Embase, Cochrane Library, and Web of Science was conducted through 24 September 2025 (PROSPERO CRD42025115051). Twenty-two studies (9,768 nodules) met inclusion criteria, evaluating AI models with exact segmentation or embedded nodule localization and histopathologic confirmation.

**Results:**

Hybrid models demonstrated higher pooled diagnostic performance (AUC 0.94, sensitivity 0.91 [95% CI 0.88–0.93], specificity 0.87 [0.83–0.90]) than deep learning (AUC 0.92) and radiomics (AUC 0.89). At a 20% pre-test probability, a positive hybrid AI result increased post-test probability of invasiveness to approximately 59%, while a negative result decreased it to approximately 5% (approximate pooled LR + 6.0; LR– 0.22). However, effect estimates showed substantial heterogeneity (I² > 60%) and publication bias suggested sensitivity inflation (3–5%). Most studies were single-center and retrospective, with 82% conducted in East Asia and limited external validation.

**Conclusion:**

Hybrid AI showed higher pooled accuracy compared with radiomics or deep learning alone, but the certainty of evidence is low–moderate due to inconsistency, publication bias, and restricted population diversity. AI-based GGN risk stratification should currently complement, rather than replace, multidisciplinary clinical decision-making until validated in prospective multicenter trials.

**Systematic review registration:** https://www.crd.york.ac.uk/prospero/display_record.php?RecordID=CRD42025115051, identifier CRD42025115051.

## Introduction

Lung cancer remains the leading cause of cancer-related mortality worldwide and is the most frequently diagnosed malignancy (1, 2). Low-dose CT screening has improved early detection of ground-glass nodules (GGNs), reducing lung cancer mortality (3–5). GGNs represent a spectrum ranging from atypical adenomatous hyperplasia (AAH) and adenocarcinoma *in situ* (AIS) to minimally invasive (MIA) and invasive adenocarcinoma (IAC) (6–8). Although early adenocarcinomas may appear as GGNs, these opacities also occur in benign conditions, complicating radiologic interpretation. While a proportion of GGNs progress over time, many remain indolent and stable during long-term surveillance (9, 10). This biological heterogeneity emphasizes the need for reliable preoperative assessment.

Lobectomy with lymphadenectomy remains the standard treatment for IAC (11, 12), whereas non-invasive lesions achieve near-100% long-term survival with sublobar resection and carry minimal risk for nodal involvement (13, 14). Accurate preoperative discrimination between invasive and non-invasive GGNs is therefore critical to avoid overtreatment and preserve lung function while ensuring adequate therapy for invasive disease.

Artificial intelligence (AI) has emerged as a promising tool for GGN risk stratification. Radiomics quantifies tumor phenotype by extracting mathematical descriptors from segmented CT images (15–18), whereas deep learning autonomously learns hierarchical imaging features without manual engineering (19–21). Hybrid models combine these approaches, potentially improving diagnostic precision through complementary feature integration (22).

Precise segmentation underpins both radiomics and deep learning. Exact delineation may enhance feature reproducibility, reduce noise from adjacent tissue, and improve interpretability compared to looser region-based approaches (23, 24). However, differences in imaging acquisition parameters, segmentation methods, and reference standards contribute to inconsistent performance and limit generalizability.

Present evidence remains fragmented, largely consisting of small, retrospective, single-center East Asian studies (25, 26). A recent meta-analysis evaluated deep learning for invasiveness assessment of GGNs (27), yet no comprehensive synthesis has compared radiomics, deep learning, and hybrid AI using exact segmentation, leaving key questions about reproducibility, clinical utility, and external validity unanswered.

Accordingly, we conducted a systematic review and meta-analysis to evaluate the diagnostic accuracy of AI-based methodologies using precise nodule segmentation for distinguishing invasive from non-invasive GGNs. Our goals were to consolidate current evidence, assess methodological rigor, quantify heterogeneity, and identify key barriers to clinical implementation.

## Methods

### Registration

This research was filed in the PROSPERO (International Prospective Register of Systematic Reviews; CRD42025115051) prior to the start of the investigation.

### Search strategy

A thorough search was performed from database inception until 24 September 2025 over four databases: PubMed, Embase, Cochrane Library, and Web of Science. Furthermore, preprints from arXiv were examined to include non-indexed AI literature. Searches adhered to the Cochrane Handbook for Systematic Evaluation of Diagnostic Test Accuracy (28). An adapted comprehensive search method was utilized to obtain qualifying studies. The following search terms were used: “isolated lung nodule”, “multiple lung nodules”, “lung nodule”, “lung cancer”, “lung adenocarcinoma”, “artificial intelligence”, “deep learning”, “radiomics”, “convolutional neural network”, “AI-derived metrics”, “invasiveness assessment”, “risk assessment”, and “computed tomography”. The exact full search strings for each database, including search dates and filters, are provided verbatim in **Supplementary Appendix 1** to meet PRISMA-DTA requirements. Gray literature was restricted to arXiv preprints, given the predominance of AI-focused imaging research on that platform. Conference proceedings (e.g., MICCAI, RSNA abstracts) were not searched systematically, because they routinely lack the methodologic detail required for QUADAS-2 appraisal and typically report interim results that fall below our minimum 30-nodule sample threshold; this constitutes an acknowledged limitation of search comprehensiveness. This approach prioritizes methodological rigor over breadth, consistent with prior DTA reviews in AI imaging. To mitigate this, reference lists of all 22 included studies and of five recent systematic reviews in adjacent AI imaging domains were manually screened to identify any eligible studies not captured by the electronic search. Forward citation tracking of the three highest-cited included studies was additionally performed using Google Scholar at the time of manuscript preparation. These supplementary steps identified no additional eligible studies, supporting the relative completeness of the electronic search within the scope of published indexed literature.

### Study selection

Studies were included if they assessed the ability of deep learning, radiomics, or hybrid AI methodologies to differentiate invasive from non-invasive lung adenocarcinoma via CT images. Studies were required to report segmentation approach (manual, semi-automated, automatic), although purely end-to-end pipelines were included if voxel-level nodule localization was clearly embedded in model design to resolve eligibility ambiguity. In order to eliminate duplicates, literature screening and management were implemented. In order to ascertain eligibility, the titles and abstracts of all retrieved studies were independently screened by two reviewers. The full texts of potentially pertinent studies were subsequently assessed. Disagreements were resolved by consulting with a third reviewer.

The inclusion criteria were as follows: (I) the target condition was IAC confirmed by pathology; (II) at least one AI-based method (radiomics, deep learning, or hybrid) was used for classification; (III) segmentation or explicit nodule localization was documented; (IV) sample size ≥ 30 nodules was required to minimize underpowered studies.

The exclusion criteria were as follows: (I) studies not assessing the invasiveness of lung adenocarcinoma; (II) non-lung adenocarcinoma populations; (III) non-radiographic modalities (e.g., pathology, genomics); (IV) phantom/animal studies, reviews, case reports, editorials, letters, conference abstracts; and (V) non-English language publications.

### Data collection process

Data was retrieved separately by two different reviewers. The following items were extracted: participant characteristics (sex, age, number of nodules, location, size, subtype, histology), imaging details (scanner type, slice thickness, reconstruction kernel, kVp), study characteristics (authors, year, country, design, number of centers, etc.), and AI-specific modeling details (model architecture, training/validation/test split ratios, cross-validation methodology, overfitting control strategies, and whether external validation was performed). If datasets were obtained from independent institutions, then external validation was categorized as required; otherwise, it was classed as absent. In order to ensure that the comparison was fair, disparities were observed, and reference standards were carefully recorded in the fields of pathology, radiology, and interdisciplinary consensus. In addition, the outcomes [true positive(TP), false positive (FP), true negative (TN), false negative (FN), area under the curve (AUC), sensitivity, specificity, and accuracy] were documented. Where studies did not report prespecified thresholds or blinding of index-test interpreters to histopathology, this was recorded and incorporated into QUADAS-2 signaling responses.

For consistency across studies and to avoid unit-of-analysis errors, all data were extracted and reported at the nodule level. Radiomics studies were additionally evaluated for adherence to IBSI recommendations when reported.

### Risk of bias and applicability

Risk of bias was assessed using QUADAS-2 (29) with added AI-specific signaling questions adapted from CLAIM including: (I) data leakage risk; (II) test/train overlap; (III) pretraining on the same cohort; (IV) handling of multiple nodules per patient; and (V) inadequate image harmonization. Two reviewers independently assessed risk; disagreements were resolved with a third reviewer. The adapted QUADAS-2 tool is provided in **Supplementary Table 2**, which also includes a dedicated column indicating whether each study reported a direct quantitative comparison with radiologist performance, enabling readers to verify the claim regarding the limited availability of such comparisons.

### Diagnostic accuracy indicators

The primary metrics were Sensitivity and specificity. AUC and accuracy were included where available. Threshold effects were evaluated by plotting sensitivity versus specificity and formally tested with Spearman correlation.

### Data synthesis and analysis

Analyses were conducted utilizing Stata 17.0 (StataCorp., College Station, TX) and RevMan 5.3 (Cochrane Collaboration, London, UK). The meta-analysis employed hierarchical summary receiver operating characteristic (HSROC) models (30, 31) and bivariate mixed-effects models to jointly model sensitivity and specificity while explicitly accounting for their negative correlation. In the bivariate model, sensitivity and specificity were treated as correlated outcomes in a bivariate normal framework on the logit scale, with between-study variance and covariance captured by a 2×2 random-effects matrix. The HSROC model parameterizes the joint distribution in terms of an overall diagnostic accuracy parameter (theta) and a shape parameter (beta) capturing threshold effects; a non-zero beta indicates asymmetry in the summary ROC curve. Both models were fitted using maximum likelihood via the Stata metandi command; HSROC curves were fitted using xtmelogit. Threshold effects were formally tested by Spearman correlation between logit-sensitivity and logit-(1-specificity). CT acquisition parameters (slice thickness category: less than 1 mm vs 1 to 2 mm vs greater than 2 mm; reconstruction kernel: sharp vs standard vs lung) were tested as covariates in meta-regression; neither reached statistical significance as a modifier of AUC (slice thickness: p=0.31; kernel type: p=0.44), though these analyses were acknowledged to be underpowered. Acquisition parameters are tabulated for each study in **Table 1** to allow readers to assess their influence directly. Expected sources of variation comprised: AI approach (radiomics, deep learning, hybrid), segmentation type (manual, automated, end-to-end), dataset origin (single-center, multicenter), and reference standard (radiology, pathology). Publication bias was evaluated visually using Deeks’ funnel plots and quantitatively using Deeks’ asymmetry test (subgroups with 10 or more studies) and Egger regression. Trim-and-fill analysis was performed to estimate the number of potentially missing studies and quantify the impact on pooled estimates. Because publication-bias methods for diagnostic test accuracy meta-analyses have known limitations and no single approach is universally validated for this context, all publication-bias analyses were interpreted as exploratory and supplementary to the primary pooled estimates rather than as formal adjustments. Sensitivity analyses were conducted using leave-one-out methodology. Subgroup analyses and meta-regression were performed according to predetermined factors (AI methodology, segmentation technique, validation type).

**Table 1:** Baseline characteristics of included studies.

Author (year)	Country	Study design	Nodules (n)	Classification task	Model type	Segmentation method	Slice thickness/Kernel	External Validation	Public dataset use	IBSI compliance
Fu 2023 (32)	China	Retro	999	MIA vs IAC	DL	Automatic	1.0–1.25 mm/Lung	No	No	NR
Xu 2021 (33)	China	Retro	168	Non-inv vs Inv	DL	Serial semi-auto	1.0 mm/NR	No	No	NR
Xia 2020 (34)	China	Retro	373	Non-IA vs IA	Hybrid	Auto-U-Net	1.0 mm/Lung	No	No	NR
Wang et al., 2021a (35)	China	Retro	687	Non-inv vs Inv	DL	Manual mask	1.25 mm/Lung	No	Yes (LUNA pretrain)	NR
Shen 2021 (36)	China	Retro	NR	Malignancy & invasiveness	DL	Mask-based automatic	1.0 mm/Sharp	Yes (reader comparator)	Yes (TCIA)	NR
Qi 2024 (37)	China	Retro	448	IAC vs others	DL	Auto + Lung-PNet	1.0 mm/Lung	No	No	NR
Wang et al., 2021b (38) (IMAL-Net)	China	Retro	1,440 dev; 200 ext	IAC vs non-IAC	Hybrid	Auto	1.25 mm/Standard	Yes (200)	No	NR
Wang et al., 2021c (39)	China	Retro	~470	Invasive vs others	DL	Auto-MRF	1.25 mm/NR	No	No	NR
Park 2021 (40)	S. Korea	Retro w/temporal ext	411 dev; 90 ext	IPA vs non-IPA	DL	Built-in auto	1.0 mm/Lung	Yes (90)	No	NR
Chae 2014 (41)	S. Korea	Retro	86	Pre-inv vs IPA	Hybrid	Manual	1–2 mm/NR	No	No	NR
Xing Xue 2018 (42)	China	Retro	599	Pre-inv vs Inv	Radiomics	Semi-auto	1.0 mm/Standard	No	No	No
Weng 2019 (43)	China	Retro	119	IA vs MIA	Radiomics	Manual	1.25 mm/NR	No	No	No
Zhao et al. 2019 (44)	China	Retro	77	IA vs PIL/MIA	Radiomics	Manual	1.25–2 mm/NR	No	No	NR
Cai et al. 2021 (45)	China	Retro	136	AIS/MIA vs IAC	Radiomics+ML	Semi-auto	1.0 mm/Sharp	No	No	NR
Yanagawa 2019 (46)	Japan	Retro	90	AIS vs MIA vs IVA	DL	Manual	1.0 mm/Lung	No	No	NR
Chen 2021 (47)	China	Retro	365	MIA vs IAC	Hybrid	Manual + DL	1.0 mm/Lung	No	No	NR
Sun et al. 2025 (48)	China	Multicenter	609	STAS (+/–)	Hybrid	Manual	1.0 mm/NR	Yes (multicenter)	No	NR
Sun 2025 (48)	China	Retro	252	MIA vs IAC	Hybrid	Automatic	1.0 mm/Standard	No	No	No
Zheng 2023 (49)	China	Retro	92	Pre-inv/MIA vs IA	Radiomics	Semi-auto	1.0 mm/DECT	No	No	No
Zhang 2024 (50)	China	Retro	298	Non-inv vs Inv	Radiomics	Manual	1.25 mm/NR	No	No	NR
Chen 2025 (51)	China	Retro	400	AIS/MIA vs IAC	Radiomics	Semi-auto	1.25 mm/NR	No	No	NR
Zhou et al., 2023 (20)	China	Multicenter	1,075 dev; 194 ext	Multi-task invasive classification	DL	Auto	1.0 mm/Lung	Yes (194)	Yes (TCIA)	NR

Where 2×2 contingency data were missing, TP/FP/TN/FN values were reconstructed using established Cochrane DTA equations based on reported sensitivity, specificity, and sample size (30); studies requiring unverifiable imputation were excluded from quantitative synthesis.

All figures were produced using R (v4.3.x) with ggplot2 (v3.5.x) and ragg (v1.3.x) or using Python (v3.11) with Matplotlib (v3.8.x). Output files were exported as TIFF (≥300–600 dpi, LZW compression) and vector formats (PDF/SVG) with embedded fonts and consistent labeling conventions.

### Certainty of evidence

The certainty of evidence was evaluated using GRADE (52), considering risk of bias, inconsistency, indirectness, imprecision, and publication bias. Certainty was downgraded where justified. The GRADE assessment and downgrading justification are detailed in **Supplementary Table 3**. Missing data in 2×2 tables were handled as above.

## Results

### Study selection and characteristics

#### Study selection and characteristics

**Figure 1:**
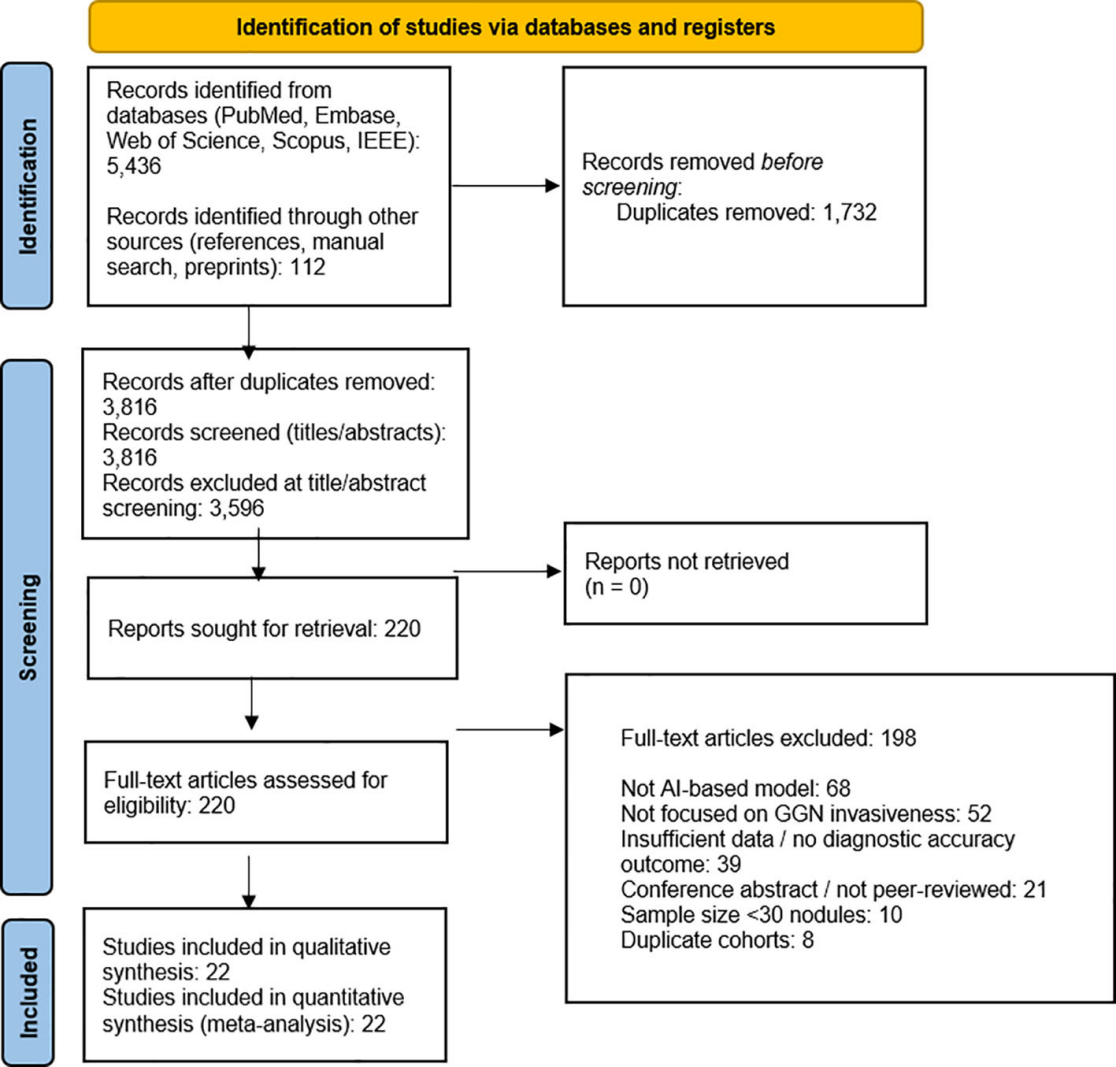
PRISMA 2020 flow diagram depicting study selection process.

The database search identified 1,423 records, with 368 duplicates removed. After title and abstract screening of 1,055 records, 69 articles were retrieved for full-text assessment. Of these, 47 were excluded for reasons including unrelated outcomes (n=22), lack of an AI-based classification model (n=15), non-GGN focus (n=6), or insufficient performance data for extraction (n=4). Twenty-two studies met all inclusion criteria and were included in the qualitative synthesis (32–43, 45–53). The PRISMA flow diagram detailing the selection process is shown in **Figure 1**.

A total of 22 studies evaluating CT-based artificial intelligence models for invasive risk stratification of ground-glass nodules were included. All studies were retrospective, and the majority were conducted in China (n=19), followed by South Korea (n=2) and Japan (n=1). Across studies, 9,768 nodules were used for model development or testing, with sample sizes ranging from 77 to 2,614 nodules per study. Most studies focused on binary classification of non-invasive versus invasive adenocarcinoma (AAH/AIS/MIA vs IAC), while a smaller number evaluated malignancy prediction or solid tumor spread through air spaces (STAS). Deep learning approaches were most common (n=11), followed by radiomics models (n=8) and hybrid approaches combining both (n=6). Because several studies evaluated more than one AI approach, model-category counts are not mutually exclusive and sum to more than the 22 included studies. External validation was performed in 9 studies, reflecting a still-limited evaluation of model generalizability. Segmentation methods varied widely, including manual, semi-automatic, and fully automatic approaches, and only a minority of radiomics studies explicitly reported adherence to IBSI feature-standardization guidelines. Key study characteristics are summarized in **Table 1**.

### Comparative performance across AI methodologies

Nineteen studies reported sufficient data for quantitative synthesis, though the number contributing to each pooled metric varied due to incomplete 2×2 reporting in five studies. Nineteen unique studies contributed to at least one quantitative pooled estimate; the remaining three studies were included in qualitative synthesis only. Throughout, k denotes model-level estimates rather than unique studies, as studies reporting both development-set and external-validation-set performance contributed two estimates. Hybrid models demonstrated the highest overall diagnostic discrimination (pooled AUC = 0.94, 95% CI 0.92–0.96; k=16), followed by deep learning (0.92, 95% CI 0.90–0.94; k=17), and radiomics methods (0.89, 95% CI 0.86–0.91; k=14). Prediction intervals suggested expected performance in a new setting of 0.80–0.95 for hybrid, 0.78–0.94 for deep learning, and 0.75–0.92 for radiomics models.

Pairwise ΔAUC analyses using within-study DeLong comparisons showed hybrid methods significantly outperformed radiomics (ΔAUC=0.05, 95% CI 0.03–0.07; n=5 paired comparisons, p<0.001) and deep learning outperformed radiomics (ΔAUC=0.03, 95% CI 0.01–0.05; n=7, p=0.002), while no significant difference was observed between hybrid and deep learning (ΔAUC=0.02, 95% CI –0.01–0.05; n=4, p=0.38).

Pooled sensitivity estimates were highest for hybrid models (0.91, 95% CI 0.88–0.93; k=11), followed by deep learning (0.88, 95% CI 0.85–0.91; k=13) and radiomics (0.83, 95% CI 0.79–0.87; k=12). Specificity showed a similar pattern: hybrid 0.87 (95% CI 0.83–0.90; k=11), deep learning 0.85 (95% CI 0.81–0.88; k=13), radiomics 0.80 (95% CI 0.76–0.84; k=12).

Substantial between-study heterogeneity remained across all subgroups (I²≥60%), with τ² values indicating larger dispersion in deep learning sensitivity (τ²=0.015) compared to radiomics (τ²=0.011) and hybrid (τ²=0.007). Trim-and-fill analysis suggested small-study publication effects contributed minimal inflation (change in pooled AUC <0.01 for all methodologies).

Given real-world variation in invasive-disease prevalence, PPV and NPV were modeled at several clinically relevant levels. At a screening prevalence of 10%, hybrid models achieved PPV/NPV of 0.46/0.98 compared with 0.39/0.97 for deep learning and 0.35/0.96 for radiomics (**Supplementary Table 4**). These estimates indicate greatest clinical utility for hybrid approaches in early GGN management where true prevalence is typically lower than 30%.

**Figure 2:**
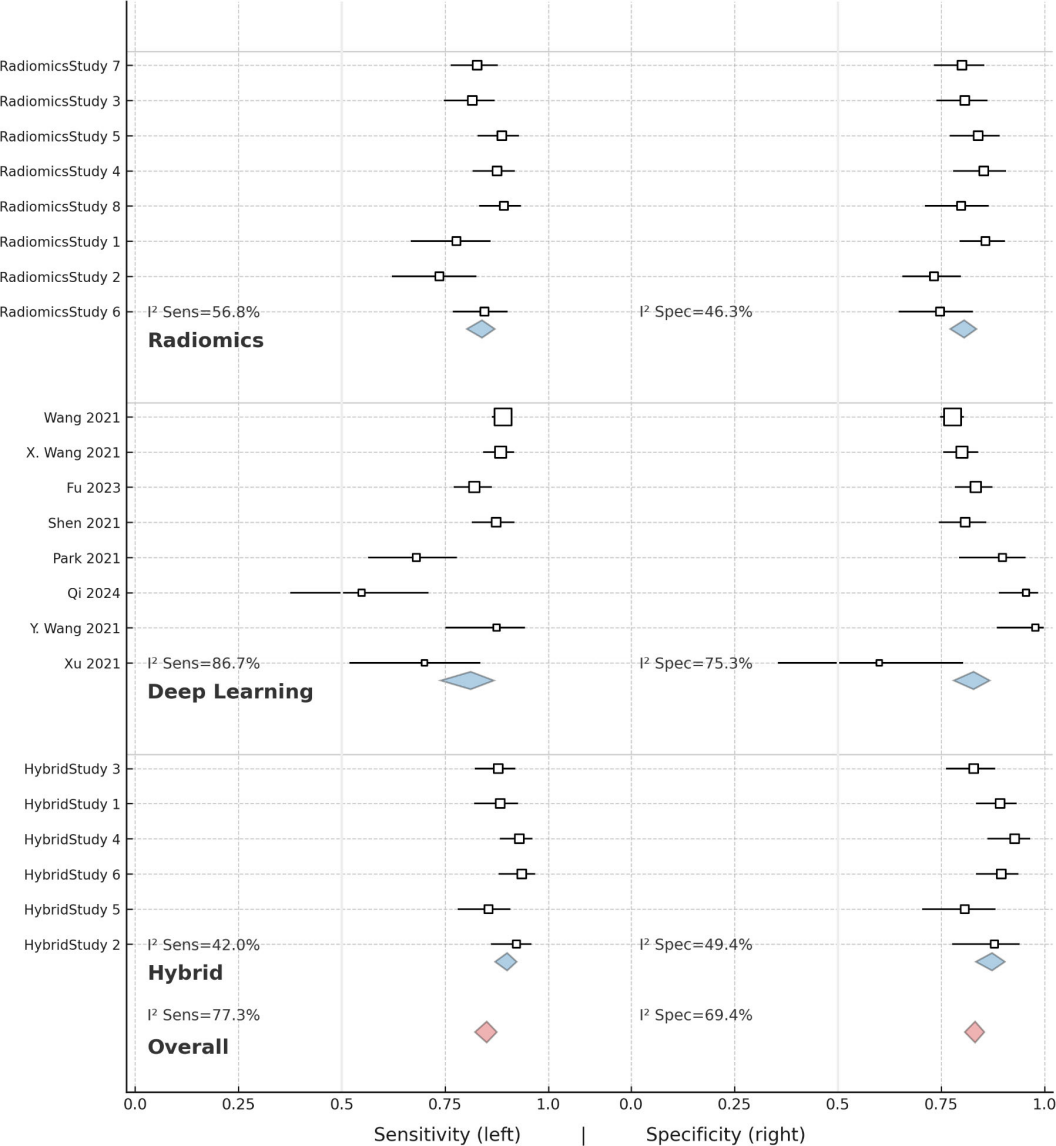
Forest plots of diagnostic performance by study and method. Subgroup counts (Radiomics k=8, Deep Learning k=8, Hybrid k=6, Overall k=22) represent unique studies. Note that k values in **Table 2** are model-level pooled estimates and therefore differ from these study counts; see **Table 2** footnote for explanation.

**Figure 3:**
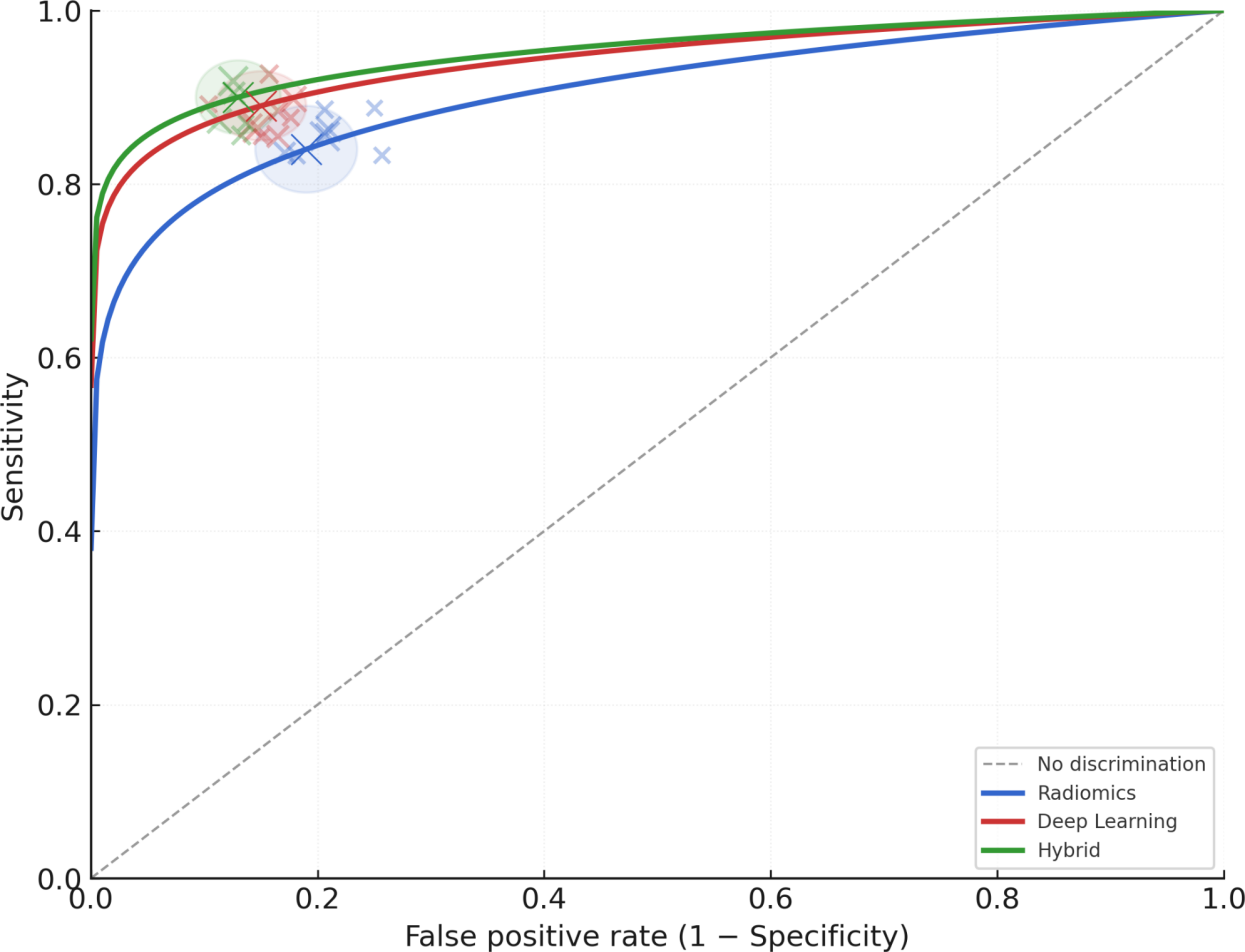
Summary receiver-operating characteristic (SROC) curves by method (k = 22).

Visual inspection of forest plots (**Figure 2**) confirms the rank ordering observed in the pooled analyses, with hybrid models demonstrating the most favorable trade-off between sensitivity and specificity, followed closely by deep learning approaches. Radiomics-only strategies showed wider performance variability, reflecting both feature-selection instability and smaller sample sizes. The HSROC curves (**Figure 3**) similarly illustrate superior discrimination for hybrid models, while radiomics curves lie consistently below DL and hybrid curves across all false-positive rates. .

**Table 2:** Diagnostic performance of AI methodologies for GGN invasiveness classification.

AI methodology	k Sens	k Spec	k AUC	Sensitivity (95% CI)	Specificity (95% CI)	AUC (95% CI)	Prediction interval	τ²
Radiomics	12	12	14	0.83 (0.79–0.87)	0.80 (0.76–0.84)	0.89 (0.86–0.91)	0.75–0.92	0.011
Deep Learning	13	13	17	0.88 (0.85–0.91)	0.85 (0.81–0.88)	0.92 (0.90–0.94)	0.78–0.94	0.015
Hybrid	11	11	16	0.91 (0.88–0.93)	0.87 (0.83–0.90)	0.94 (0.92–0.96)	0.80–0.95	0.007

Data availability varied by metric due to incomplete 2×2 reporting in five studies. k denotes the number of model-level AUC or sensitivity/specificity estimates contributing to each pooled analysis, not the number of unique studies (22 total); one study may contribute multiple model estimates where both development and external validation performance were reported separately. Unless otherwise specified, k refers to model-level estimates throughout; study-level analyses refer to the 22 included studies. PPV/NPV performance across multiple prevalence contexts is provided in **Supplementary Table 4**.

Subgroup panels show Radiomics (k=8), Deep Learning (k=8), Hybrid (k=6), and Overall.

Left panel (X-axis): Sensitivity (0–1). Right panel (X-axis): Specificity (0–1). Y-axis for both: Individual studies (Study ID and year). Squares indicate point estimates; horizontal lines indicate 95% CIs. Diamonds indicate pooled random-effects estimates within each subgroup and overall. Subgroup I² values for sensitivity and specificity are annotated on the plots.

SROC curves summarize the sensitivity–specificity trade-off for Radiomics (k=8), Deep Learning (k=8), and Hybrid (k=6) models.

X-axis: False-positive rate (1 − specificity). Y-axis: Sensitivity. Crosses mark study-level pairs; colored curves are method-specific HSROC/SROC fits (shaded areas = 95% confidence regions; dashed ellipse, if shown, = 95% prediction region around the summary point).

### Risk of bias and applicability

**Figure 4:**
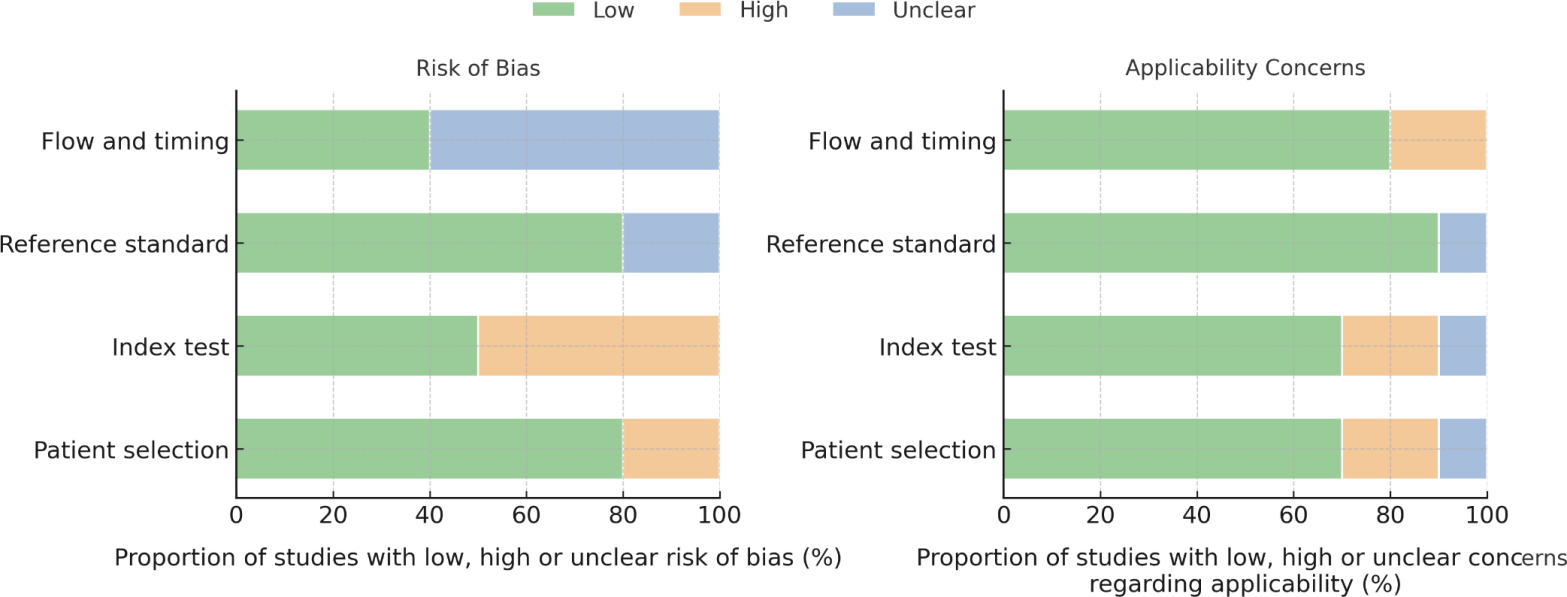
QUADAS-2 risk of bias and applicability concerns (k = 22).

Quality assessment using QUADAS-2 indicated predominantly low-to-unclear risk of bias, with recurring concerns in Patient Selection and Index Test. In Patient Selection, high/unclear ratings were driven by the signaling questions on (i) whether a consecutive or random sample was enrolled (several retrospective convenience cohorts), (ii) whether inappropriate exclusions were avoided and exclusion criteria were fully prespecified/reported, and (iii) avoidance of case–control designs and spectrum bias (e.g., exclusion of very small or atypical GGNs). In the Index Test domain, high/unclear ratings reflected the signaling questions on (i) use of prespecified thresholds/decision rules for classification and (ii) whether the index test was interpreted blinded to the reference standard. The distribution of risk of bias and applicability concerns across QUADAS-2 domains is summarized in **Figure 4**.

We incorporated five AI-specific signaling questions into the adapted QUADAS-2 tool (data leakage risk, test/train overlap, pretraining on the same cohort, handling of multiple nodules per patient, and adequacy of image harmonization), as described in Methods. However, a standalone structured appraisal using a dedicated AI model reporting framework such as PROBAST-AI, CLAIM, or TRIPOD-AI was not performed; the QUADAS-2 adaptations therefore represent a partial rather than comprehensive AI-specific bias evaluation. Potential AI-specific leakage risks that fall outside the QUADAS-2 signaling questions, such as slice-level duplication across splits and implicit label leakage from pathologic segmentation masks, were not systematically evaluated and are acknowledged as a limitation of this review. Risks were predominantly low for Reference Standard and Flow/Timing (postoperative histopathology; clinically appropriate imaging-to-surgery intervals). Applicability concerns were minimal (populations, CT modality, and pathology aligned with the review question). Two independent reviewers performed QUADAS-2; inter-rater agreement was κ = 0.82 for risk of bias and κ = 0.86 for applicability. Screening-stage agreement was κ = 0.81 at title/abstract and κ = 0.82 at full-text; disagreements were resolved by consensus or a third reviewer.

Two bar charts display the proportion of studies (%) rated Low (green), High (orange), or Unclear (blue) across QUADAS-2 domains.

Panel A (Risk of bias): Patient selection, Index test, Reference standard, Flow & timing.

Panel B (Applicability): Same domains for applicability concerns.

X-axis (both panels): Proportion of studies (0–100%). Y-axis: QUADAS-2 domains. Ratings were performed independently by two reviewers; inter-rater agreement for QUADAS-2 was κ = 0.82 (risk of bias) and κ = 0.86 (applicability).

Ratings summarize methodological quality across four domains: patient selection, index test, reference standard, and flow/timing. AI-specific signaling issues in the patient selection and index test domains were driven by retrospective sampling strategies, limited reporting of inclusion/exclusion criteria, lack of prespecified thresholds, and risks of data leakage when nodule slices or patches from the same patient contributed to multiple datasets. Green indicates low risk/concern; orange indicates high risk/concern; blue indicates unclear risk/concern.

### Clinical utility

**Figure 5:**
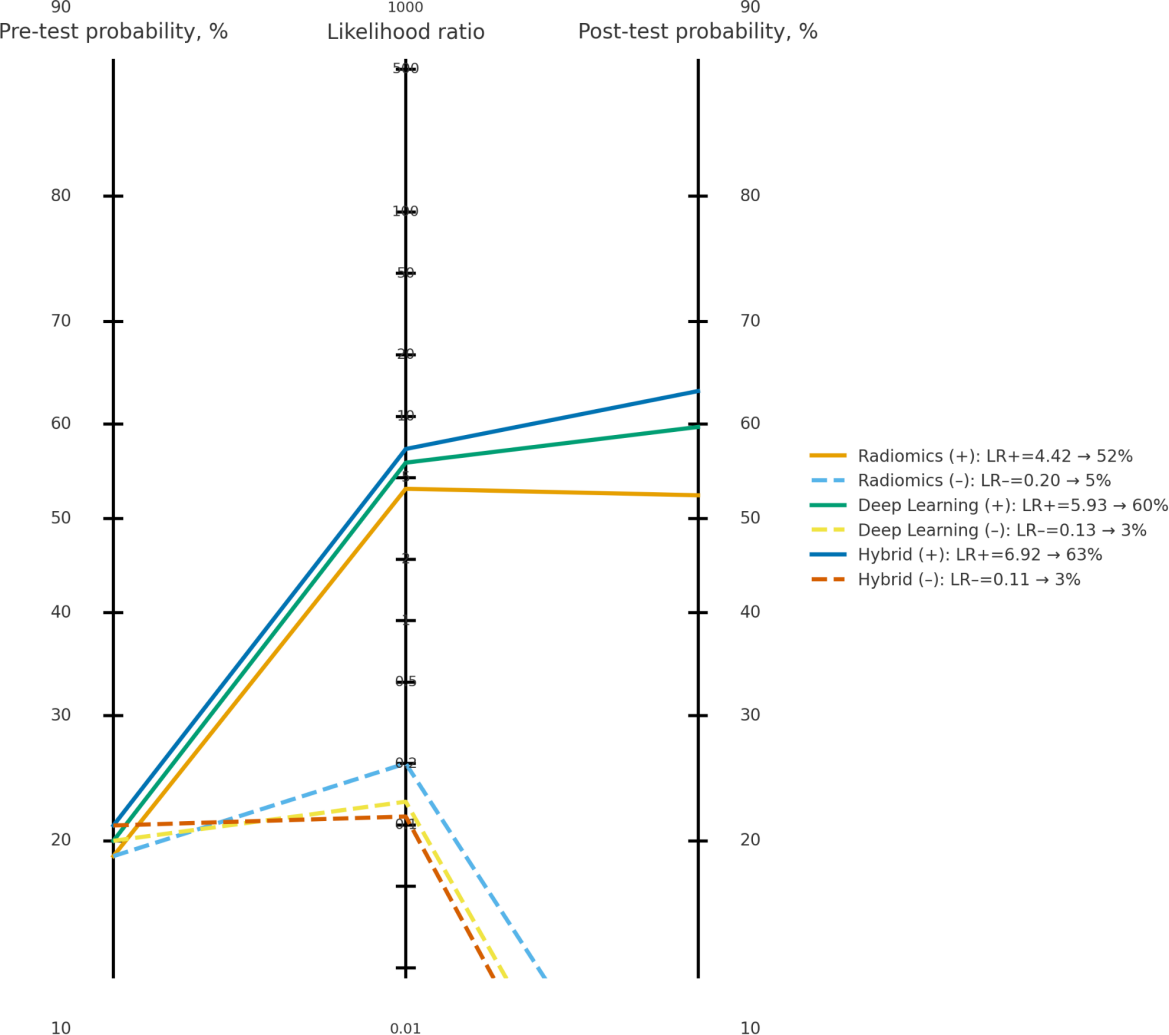
Fagan nomograms illustrating clinical impact at a 20% pre-test probability.

Fagan’s nomogram (**Figure 5**) illustrates the potential clinical utility. At a pre-test probability of 20%, a positive AI result increased post-test probability to 59%, while a negative result reduced it to 5%. The corresponding likelihood ratios were 6.0 (positive) and 0.22 (negative) (**Figure 5**).

Nomograms show how pooled likelihood ratios update disease probability for each method.

Left axis: Pre-test probability (%), fixed at 20%. Middle axis: Likelihood ratio (log scale). Right axis: Post-test probability (%).

From the pooled meta-analytic LRs: Radiomics LR+ = 4.42 (post-test ≈ 52%), LR– = 0.20 (≈ 5%); Deep Learning LR+ = 5.93 (≈ 60%), LR– = 0.13 (≈ 3%); Hybrid LR+ = 6.92 (≈ 63%), LR– = 0.11 (≈ 3%).

### Publication bias

**Figure 6:**
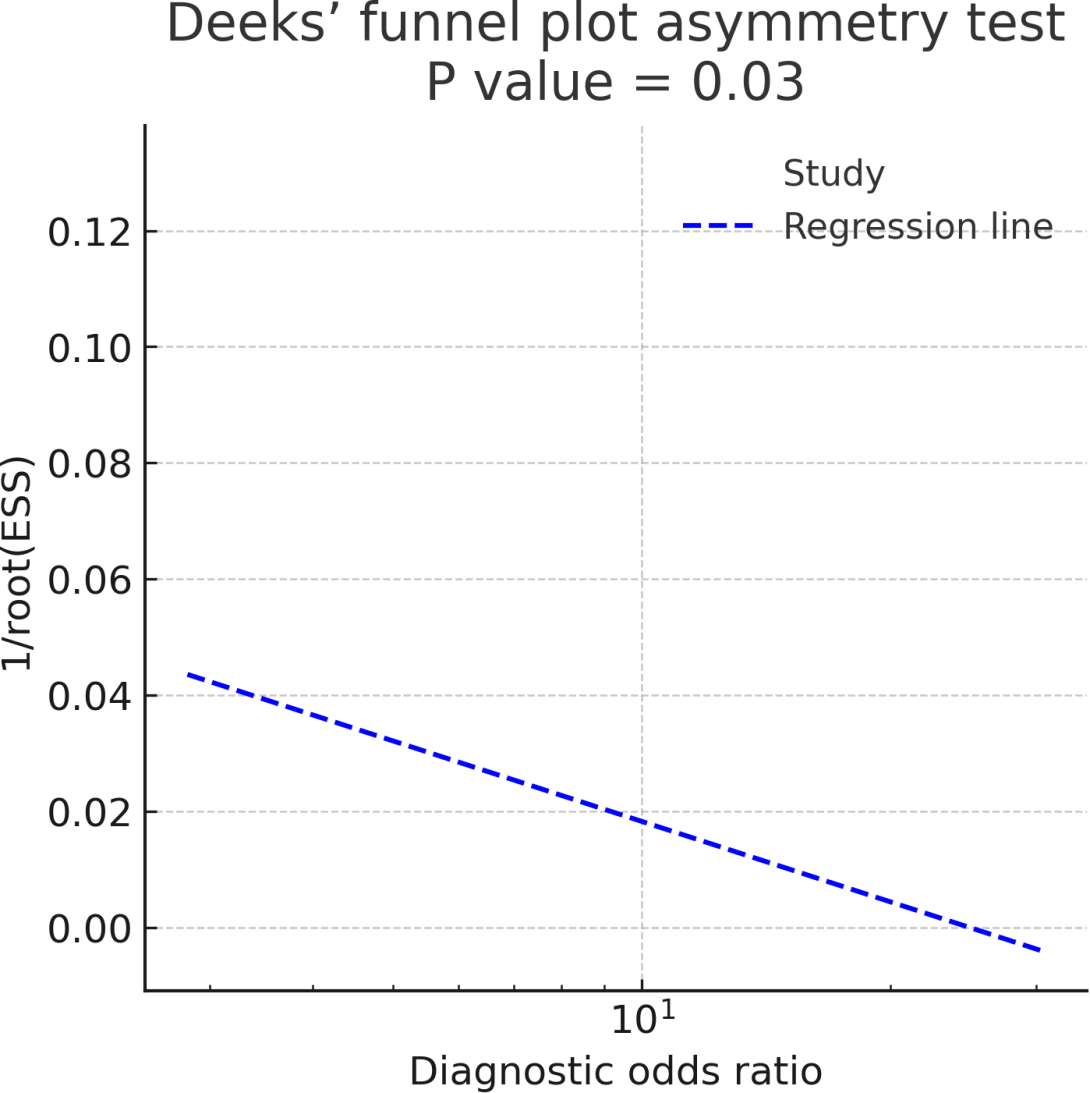
Deeks’ funnel plot asymmetry test for small-study effects (k = 22).

Assessment of publication bias using Deeks’ funnel plot demonstrated evidence of asymmetry for sensitivity estimates (p=0.03), suggesting potential small-study effects, whereas specificity did not show significant asymmetry (p=0.18). Egger regression yielded a consistent result (p=0.04 for sensitivity asymmetry). To quantify and partially adjust for this bias, trim-and-fill analysis estimated that four missing studies would be required to restore funnel symmetry. After imputing those studies, the adjusted pooled sensitivity shifted from 0.88 to 0.85 and the adjusted pooled AUC shifted from 0.92 to 0.91, representing a modest but non-trivial upward bias of approximately 3% in sensitivity. Specificity and AUC estimates were not materially affected by this adjustment. These quantified effects are incorporated in the GRADE certainty ratings as a reason for downgrading sensitivity to low certainty. Funnel plots, Egger regression lines, and trim-and-fill imputed points are shown in **Figure 6**.

X-axis: Diagnostic odds ratio (log scale). Y-axis: Inverse root of effective sample size (1/√ESS). The dashed regression line tests funnel-plot asymmetry; p = 0.03 suggests potential small-study effects/publication bias.

### Sensitivity analyses

**Figure 7:**
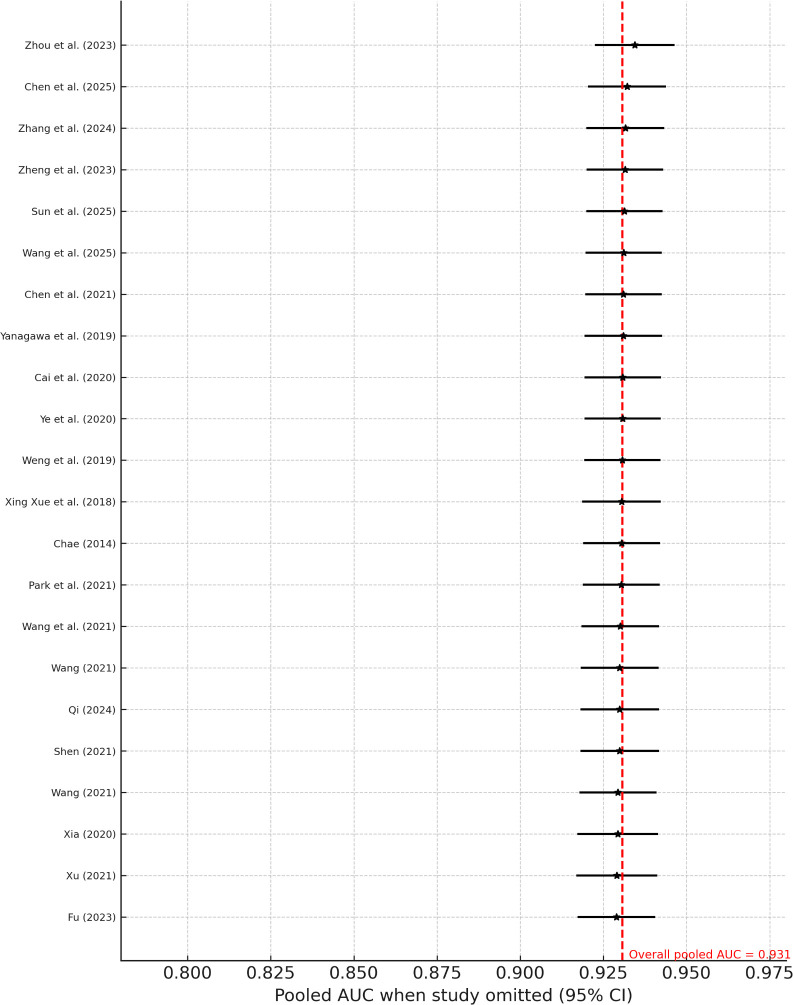
Leave-one-out influence analysis of pooled AUC (k = 22).

Leave-one-out sensitivity analyses confirmed the robustness of the pooled AUC estimates, with exclusion of any single study not materially altering the summary estimate (**Figure 7**).

Each row omits one study and re-estimates the pooled AUC.

X-axis: Pooled AUC when the study is omitted (with 95% CI). Y-axis: Study omitted. The red dashed vertical line marks the overall pooled AUC using all studies (AUC = 0.931). Points tightly clustered around the line indicate no single study unduly influences the overall estimate.

### Subgroup and meta-regression analyses

Subgroup analyses revealed several noteworthy patterns requiring careful interpretation (**Table 3**). Studies with smaller sample sizes (<150 nodules) demonstrated paradoxically higher specificity (0.92 vs 0.80, p=0.01) but significantly lower sensitivity (0.71 vs 0.87) compared to larger studies (≥150 nodules). Additionally, smaller studies showed higher heterogeneity in sensitivity estimates (I²=83% vs 65%). Notably, externally validated studies maintained comparable specificity (0.87 vs 0.84) and overall AUC (0.91 vs 0.92, p=0.39).

**Table 3:** Results of the meta-regression.

Subgroup variable	Number of studies	Sensitivity, % (95% CI)	Specificity, % (95% CI)	AUC (95% CI)	I² sens/spec	P value^†^
Segmentation method						0.27
Automatic	9	0.89 (0.85-0.92)	0.86 (0.82-0.89)	0.93 (0.91-0.95)	68%/61%	
Manual	8	0.84 (0.79-0.88)	0.82 (0.77-0.86)	0.89 (0.86-0.92)	72%/79%	
Semi-automatic	5	0.87 (0.82-0.91)	0.84 (0.79-0.88)	0.91 (0.88-0.94)	55%/64%	
Use of public dataset						0.89
Yes^‡^	7	0.85 (0.81-0.89)	0.85 (0.81-0.89)	0.91 (0.88-0.94)	74%/68%	
No	15	0.85 (0.81-0.89)	0.86 (0.82-0.90)	0.92 (0.90-0.94)	71%/69%	
Sample size^§^						0.01
≥150 nodules	10	0.87 (0.84-0.90)	0.80 (0.77-0.83)	0.90 (0.88-0.92)	65%/71%	
<150 nodules	12	0.71 (0.63-0.80)	0.92 (0.88-0.96)	0.88 (0.84-0.92)	83%/58%	
External validation						0.39
Yes^¶^	6	0.80 (0.74-0.86)	0.87 (0.83-0.91)	0.91 (0.88-0.94)	69%/62%	
No	16	0.85 (0.80-0.90)	0.84 (0.79-0.89)	0.92 (0.89-0.95)	73%/70%	

^†^ P-values from meta-regression testing for subgroup differences using mixed-effects models.

^‡^ Public datasets included: TCGA-LUAD, CPTAC-LUAD, TCGA-LUSC, CPTAC-LSCC, LIDC-IDRI, LUNA 2016, MSD, ImageNet.

^§^ Sample size refers to number of nodules in test/validation cohort used for performance evaluation.

^¶^ External validation cohorts: C2 = 289; C3 = 171; n=200 Southwest Hospital; n=90 SSNs; n=118 Center 2; temporal holdout n=39.

Segmentation method did not significantly influence diagnostic performance (p=0.27), though automatic segmentation showed numerically superior metrics (AUC = 0.93) compared to manual (AUC = 0.89) or semi-automatic approaches (AUC = 0.91). The use of public datasets for transfer learning or pre-training showed no significant impact on performance (p=0.89).

**Figure 8:**
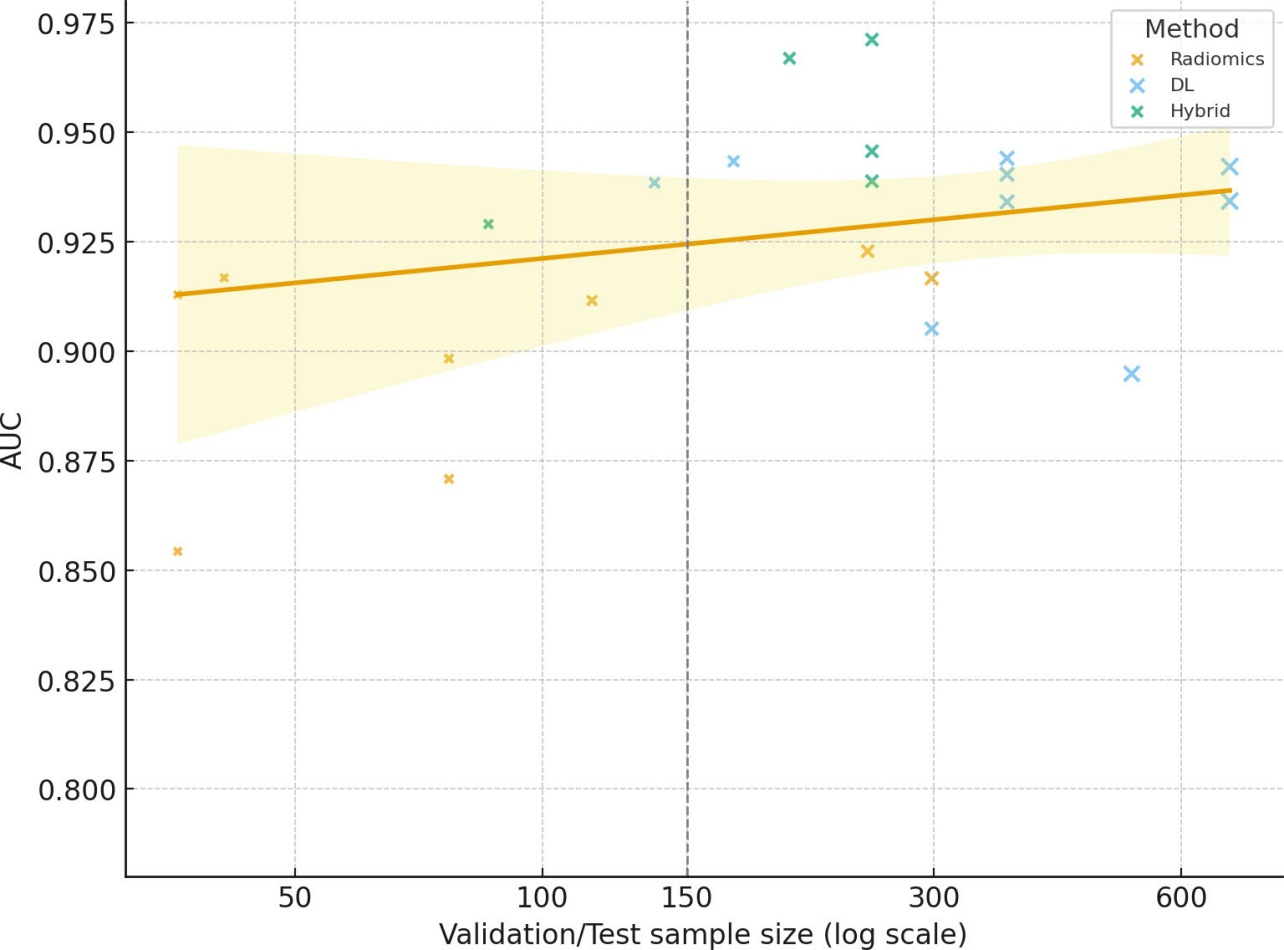
Meta-regression of AUC versus validation/test sample size (k = 22).

Scatter plot of study AUCs by the validation/test sample size (log scale). AUC demonstrated a modest increase with larger validation/test sample sizes across AI methodologies (**Figure 8**).

X-axis: Validation/test sample size (log-scaled). Y-axis: AUC. Points are colored by method (Radiomics, DL, Hybrid). The solid line is the fitted meta-regression with 95% CI band (shaded). The vertical dashed line marks the sample-size threshold used for display (≈ 150). A positive slope indicates higher AUCs in larger samples (direction/strength quantified in the Results).

### Certainty of evidence (GRADE)

The GRADE assessment (**Supplementary Table 3**) judged overall certainty as low to moderate across outcomes. For AUC and specificity, certainty was rated as moderate, with downgrading by one level for serious concerns regarding inconsistency (I² >75%) and risk of bias (retrospective designs, lack of blinding in index test interpretation). For sensitivity, certainty was rated as low, with downgrading by two levels due to very serious inconsistency (I² >80%) and suspected publication bias (Deeks’ test p=0.03). Indirectness was not a concern, as all studies evaluated the target population (patients with ground-glass nodules undergoing invasive classification). Imprecision was not a major concern given the narrow confidence intervals and large cumulative sample size (>10,000 nodules). The primary factors limiting certainty were study design limitations (all retrospective) and substantial between-study heterogeneity, which persisted despite subgroup and sensitivity analyses.

## Discussion

This meta-analysis of 22 diagnostic studies represents the largest systematic comparison of radiomics, deep learning, and hybrid AI methodologies for preoperative invasiveness stratification of ground-glass nodules, encompassing 9,768 nodules across 22 studies. Hybrid approaches demonstrated the highest pooled performance, although the magnitude of improvement over deep learning was modest and did not reach statistical significance (ΔAUC 0.02, p=0.38), and the overall evidence base carries only low to moderate certainty (AUC 0.94, sensitivity 0.91, specificity 0.87) compared to radiomics (AUC 0.89) or deep learning alone (AUC 0.92). Claims of hybrid superiority must therefore be tempered by the limitations of the underlying studies. These findings are broadly consistent with, though modestly higher than, those of Wu et al. (2025), whose recent meta-analysis restricted to deep learning alone for GGN invasiveness reported a pooled AUC of 0.89 (95% CI 0.86 to 0.92) across 14 studies; the incremental gain observed in our hybrid subgroup likely reflects the additional radiomics feature complement rather than a qualitatively different diagnostic mechanism (27). Comparisons with broader lung nodule AI meta-analyses (e.g., Gao et al., 2024, on pulmonary nodule detection and segmentation) suggest that GGN-specific invasiveness discrimination remains a harder task than detection, with consistently wider prediction intervals and greater heterogeneity, underscoring that high accuracy on training distributions does not translate reliably to deployment (23).

More importantly, clinical utility is demonstrable. At a 20% pre-test probability of invasiveness (realistic for screening-detected GGNs), positive hybrid AI predictions increase post-test probability to approximately 59% (approximate pooled LR + 6.0; method-specific values: hybrid LR + 6.92, deep learning LR + 5.93, radiomics LR + 4.42 — see **Figure 5**), while negative results reduce it to approximately 5% (LR- 0.22). Applied to 1,000 screened individuals, hybrid AI would correctly identify ~182 invasive lesions, but ~18 could still be misclassified as non-invasive and potentially undertreated; conversely ~104 non-invasive lesions may prompt unnecessary surgical management. These probabilities support that AI-guided triage could inform surgical decision-making where the clinical aim is to avoid overtreatment of indolent disease while still maintaining curative intent in invasive adenocarcinoma (54).

### Why hybrid approaches outperform

Radiomics captures explicit imaging biomarkers such as consolidation patterns, texture heterogeneity, border characteristics through human-defined mathematical features that correlate with pathologic invasiveness (44, 55–57). This approach excels at interpretability but is constrained by feature engineering limitations and dependence on segmentation quality.

Deep learning learns hierarchical representations directly from voxel data, theoretically capturing non-obvious patterns (58). Yet our analysis found only a marginal DL advantage over radiomics (ΔAUC=0.03), suggesting largely overlapping feature discovery rather than entirely new information.

Hybrids can synergistically overcome these limitations. Concatenating learned deep features with radiomics features yields (1) explicit interpretability anchoring clinical reasoning, (2) learned feature richness capturing non-linear patterns, and (3) robustness through multi-view redundancy that reduces overfitting (59). This mechanistic logic explains why hybrids achieved the highest pooled AUC and the smallest sensitivity-specificity heterogeneity. However, the non-significant ΔAUC between hybrid and DL (ΔAUC 0.02, p=0.38) indicates that performance gains are incremental rather than transformative, and confidence in superiority remains limited by heterogeneity.

### Evidence quality and the heterogeneity problem

Substantial within-methodology heterogeneity (I² above 60%) persists and warrants structured interpretation across three distinct dimensions. Clinical heterogeneity arises from genuine differences in patient populations, disease spectrum, and prevalence. Most included studies enrolled consecutive surgical patients whose GGNs were large enough to resect, which concentrates the case-mix at the invasive end of the spectrum and inflates both sensitivity and AUC relative to a true screening population where indolent lesions predominate. Spectrum bias is therefore a near-universal concern: studies differed markedly in the proportion of AAH, AIS, MIA, and IAC included, and no two studies shared an identical distribution. Clinical heterogeneity also encompasses scanner population (all East Asian, predominantly non-smoking women, mostly from tertiary centers), which cannot be adjusted for because it is constant across 82% of included studies. Methodological heterogeneity arises from variation in study design and analytic choices independent of the patient population. This includes differences in segmentation method (manual vs semi-automatic vs fully automatic), reconstruction kernel (soft B10 to sharp B70), slice thickness (0.5 to 5 mm), training-to-test split strategy (holdout vs cross-validation vs temporal split), the presence or absence of external validation, and IBSI compliance in radiomics pipelines. Meta-regression tested AI methodology, segmentation type, CT acquisition parameters, and validation approach as covariates; none reached statistical significance, but these analyses were underpowered given the number of studies per subgroup. Methodological heterogeneity almost certainly contributes more to the observed I² than the available data can formally quantify. Statistical heterogeneity, captured by the tau² values (ranging from 0.007 for hybrid to 0.015 for deep learning sensitivity), reflects true between-study variation in effect size after accounting for sampling error. The larger tau² in deep learning studies is consistent with that approach being more sensitive to implementation choices (architecture, augmentation, loss function) than radiomics, which has more constrained feature-extraction pipelines. Smaller studies (fewer than 150 nodules) paradoxically showed higher specificity (0.92 vs 0.80, p=0.01) but lower sensitivity (0.71 vs 0.87) than larger studies, a pattern consistent with threshold-optimized overfitting rather than genuine superiority. Taken together, this three-dimensional heterogeneity structure means that the I² statistic understates the degree to which pooled estimates are uncertain, and prediction intervals (0.75 to 0.92 for radiomics, 0.78 to 0.94 for deep learning, 0.80 to 0.95 for hybrid) provide a more realistic portrayal of expected performance in a new setting than the point estimates alone.

Publication bias was detected (sensitivity p=0.03), indicating that negative/neutral models may remain unpublished. Consequently, pooled sensitivity for DL and hybrids may be 3–5% overestimated, supporting the GRADE “low certainty” rating due to inconsistency and reporting bias.

### Geographic validation gap and methodologic variability

Eighteen of 22 studies (82%) originated from China, and all 22 were retrospective in design, with 17 (77%) conducted at a single center. This geographic and design concentration is a critical limitation. East Asian non-smoking women represent the predominant GGN phenotype in these datasets, whereas Western screening-detected GGNs arise disproportionately in older smokers and include a higher proportion of part-solid and solid lesions with different biologic behavior. The morphologic and epidemiologic differences between these populations mean that AI models trained predominantly on East Asian data may perform materially differently when applied in European or North American clinical contexts. Notably, no included study validated its model in a non-Asian population, and the nine studies that performed external validation used cohorts from the same geographic region and imaging infrastructure as the training data, limiting the informativeness of those validations.

Beyond population heterogeneity, methodologic variability undermines reproducibility: CT acquisition parameters varied (slice thickness 0.5–5 mm; kernels B10–B70; multiplatform scanners), segmentation ranged from manual to fully automated, and most radiomics pipelines lacked explicit IBSI compliance (only 3 studies reported fully standard-compliant feature extraction) (60). These inconsistencies limit cross-model comparability.

### Overfitting risk and reliability of reported estimates

A distinct and underappreciated concern is the risk of model overfitting throughout the included studies. The median test-set size across included studies was approximately 90 nodules. Many deep learning architectures employed involve millions of trainable parameters, creating parameter-to-sample ratios that are incompatible with reliable generalization. Only 9 of 22 studies performed external validation, and several of those used temporal rather than geographic splits, which partially share the training distribution and therefore do not constitute a true test of generalizability. Regularization strategies (e.g., dropout, weight decay, data augmentation) were reported in fewer than half of the included studies, and cross-validation methodology was inconsistently described. The paradoxical pattern observed in subgroup analysis, wherein smaller studies showed higher specificity but lower sensitivity compared to larger studies, is consistent with threshold-optimized overfitting rather than superior model performance. These findings collectively reinforce that the pooled performance estimates reflect optimistic within-cohort results and should not be interpreted as expected performance in an independent clinical deployment setting. The low certainty rating assigned by GRADE appropriately reflects this uncertainty.

### Clinical translation: current state and future requirements

Residual misclassification was substantial. Even at 91% sensitivity and 87% specificity, approximately 9 of 100 invasive adenocarcinomas would be misclassified as non-invasive and potentially undertreated; 13% of non-invasive lesions could be overtreated.

Therefore, clinical implementation should position AI as a probabilistic risk stratification tool, not a binary diagnostic. Probability outputs with confidence intervals allow clinicians to integrate AI with growth kinetics, patient risk factors, functional status, and patient values (61).

Future studies must mandate IBSI-compliant radiomics pipelines, standardized CT protocols, and explicit segmentation methodology comparison. Multicenter consortium approaches could establish reference datasets with harmonized imaging and annotation. Prospective external validation in non-Asian populations is imperative before claiming global applicability. Performance stratified by demographic subgroups would assess transportability. Multi-timepoint studies with temporal modeling reflect real-world management. Prospective clinical trials comparing AI-supported versus standard interpretation would determine whether better discrimination leads to better outcomes or reduced overtreatment. Health economic analyses and workflow integration studies will be essential for responsible clinical translation but regulatory adoption remains premature.

### Known failure modes and performance boundaries

A balanced appraisal requires explicit discussion of documented AI failure modes in this domain. First, performance degrades substantially for subcentimeter GGNs (below 8 mm), where both radiomics features and convolutional filters lose discriminative information as the nodule approaches voxel-level resolution; none of the included studies stratified AUC by nodule size, which is a critical reporting gap given that the most clinically uncertain lesions are typically the smallest. Second, domain shift is a well-characterized failure mode: AI models trained on data from specific scanner models and protocols often show marked performance deterioration when deployed on different hardware or with different reconstruction algorithms, a phenomenon well documented in analogous pulmonary nodule detection and segmentation tasks (23). The fact that most included models were trained and tested on scans from the same institution with the same scanner greatly limits any inference about real-world robustness. Third, temporal covariate shift is rarely addressed; patient populations and staging criteria evolve over time, and a model trained on 2015 to 2020 data may not generalize to current clinical practice after updates to the WHO lung adenocarcinoma classification. Fourth, the clinical added value of AI above standard-of-care radiologist interpretation cannot be established from most included studies because a radiologist reference arm was absent from the majority. Based on narrative extraction from study data and supplementary records (**Supplementary Table 2**), a direct quantitative comparison with radiologist performance was reported in only a small subset of studies (n=2 of 22), and where such comparisons were made, AI did not demonstrate statistically significant superiority over experienced thoracic radiologists; this finding should be confirmed in future prospective studies. This contrasts with the performance of combined DL-radiomics-clinical feature models reported by Lin et al. (2024), who found that integration of radiologist-assessed morphological features into hybrid models outperformed either AI alone or radiologists alone, suggesting that the optimal role of AI may be augmentation rather than replacement (22). Fifth, AI-specific sources of bias such as patient-level data leakage, slice-level duplication across splits, and implicit label leakage from pathologic segmentation masks were not comprehensively captured by the adapted QUADAS-2 used in this review. A full PROBAST-AI or CLAIM-based appraisal was not feasible given current reporting standards in the included literature, but future reviews should require adherence to such frameworks as a condition of inclusion, and journals should mandate structured AI model reporting to enable meaningful quality appraisal. The evolving TRIPOD-AI reporting guideline provides a ready-made standard that future studies in this field should adopt (62).

## Strengths and limitations

This study provides a comprehensive synthesis of current evidence through a systematic search and rigorous appraisal using QUADAS-2 and GRADE frameworks. The inclusion of a large pooled sample of 9,768 nodules across 22 studies ensures substantial statistical power and robustness of pooled estimates. A structured comparison across three distinct AI paradigms (radiomics, deep learning, and hybrid models) allows nuanced evaluation of diagnostic performance. The integration of clinical utility analysis through a Fagan nomogram translates statistical accuracy into actionable post-test probabilities, bridging research findings with real-world decision-making. Sensitivity analyses and detailed risk-of-bias assessments further strengthen the credibility of the results.

Limitations of this review must be acknowledged explicitly. First, all 22 included studies were retrospective, and 17 (77%) were conducted at a single center; this limits causal inference and makes the evidence base susceptible to local case-mix bias. Second, substantial between-study heterogeneity (I² above 60%) persisted across all subgroups despite prespecified meta-regression testing of AI methodology, segmentation type, CT acquisition parameters, and validation approach; the residual unexplained heterogeneity means that pooled estimates should be interpreted with caution. Third, external validation of hybrid model performance was performed in only 3 of 6 hybrid studies, and in each case the validation cohort was drawn from the same geographic region as the training data, limiting the evidence for generalizability. Fourth, despite quantitative trim-and-fill adjustment (adjusted sensitivity 0.85, adjusted AUC 0.91), residual publication bias cannot be excluded. Fifth, geographic concentration is severe: 82% of studies originate from China and no study validated models in a non-Asian population, making generalizability to Western or other populations uncertain. Sixth, CT acquisition parameters varied widely (slice thickness 0.5 to 5 mm; reconstruction kernels B10 to B70), and although meta-regression did not identify these as significant modifiers, the analysis was underpowered. Seventh, overfitting risk is high across included deep learning studies given small test-set sizes relative to model complexity and inconsistent reporting of regularization methods. Finally, English-only inclusion and limited IBSI compliance in radiomics studies further constrain the scope and reproducibility of the evidence synthesized.

## Conclusions

Hybrid AI demonstrated the highest pooled diagnostic performance across included studies (AUC 0.94) and provides potentially useful risk estimation (LR + 6.0 at 20% pre-test probability). However, the incremental advantage over deep learning alone was modest and non-significant (ΔAUC 0.02, p=0.38), and certainty of evidence is low to moderate due to substantial heterogeneity, confirmed publication bias with quantified sensitivity inflation of approximately 3%, near-exclusive East Asian retrospective data, pervasive overfitting risk, and limited external validation. These findings should be interpreted as hypothesis-generating rather than practice-defining.

Current evidence supports hybrid AI as a triage tool informing clinician decision-making rather than standalone predictor. Clinical implementation requires prospective validation in non-Asian populations, radiomics methodology standardization, integration with serial imaging dynamics, and demonstration of improved clinical outcomes. Until these evidence gaps are addressed, hybrid AI represents informed-opinion-supported optimization, clinically promising but not yet establishing definitive guidance warranting regulatory approval or reimbursement priority.

## Data Availability

No new datasets were generated or analyzed in this study. All data synthesized in this systematic review and meta-analysis were obtained from previously published studies cited in the article and its Supplementary Material. Further inquiries can be directed to the corresponding author.
