# Supplementary material for: Comparative diagnostic accuracy of radiomics, deep learning, and hybrid AI for invasiveness stratification of pulmonary ground-glass nodules: a systematic review and meta-analysis
Source: Front Oncol. 2026 Jul 24;16:1752652. doi: 10.3389/fonc.2026.1752652 (PMC13447058; doi:10.3389/fonc.2026.1752652)
Supplement: Supplementary file 1 [file Table1.docx]

Supplementary Table 1: QUADAS 2

| Author (year) | QUADAS-2: Patient selection | QUADAS-2: Index test | QUADAS-2: Reference | QUADAS-2: Flow/Timing | Overall risk of bias | Applicability (Patient) | Applicability (Index test) | Applicability (Reference) |
| --- | --- | --- | --- | --- | --- | --- | --- | --- |
| Fu, 2023 | High | Low | High | Low | High | Low | Low | Low |
| Xu, 2021 | High | Low | Low | Low | High | Low | Low | Low |
| Xia, 2020 | High | Low | High | Low | High | Low | Low | Low |
| Wang, 2021 | High | Low | Low | Low | High | Low | Low | Low |
| Shen (2021) | High | Low | Low | Low | High | High | Low | Low |
| Qi (2024) | High | High | Low | High | High | High | High | High |
| Wang (2021) | Low | Low | Low | High | Low | Low | Low | Low |
| Wang et al. (2021) | High | High | High | High | High | High | High | Low |
| Park et al. (2021) | High | High | Low | High | High | High | Low | Low |
| Chae (2014) | High | High | High | Low | High | High | High | High |
| Xing Xue et al. (2018) | High | High | High | High | High | High | High | Low |
| Weng et al. (2019) | High | High | Low | Low | High | High | High | Low |
| Ye et al. (2020) | High | High | Low | High | High | High | High | Low |
| Cai et al. (2020) | High | High | Low | Low | High | High | High | Low |
| Yanagawa et al. (2019) | High | High | High | High | High | High | High | High |
| Chen et al. (2021) | High | Low | Low | Low | High | High | High | Low |
| Wang et al., 2025 | High | High | High | High | High | High | Low | Low |
| Sun et al. (2025) | High | High | Low | High | High | High | High | Low |
| Zheng et al. (2023) | High | High | Low | High | High | High | High | Low |
| Zhang et al. (2024) | High | High | Low | Low | High | High | High | Low |
| Chen et al. (2025) | High | High | Low | Low | High | High | High | Low |
| Zhou et al. (2023) | High | High | High | High | High | High | High | High |

Supplementary Table S2. Study-level GRADE assessment for AI models classifying malignancy of ground-glass nodules

| Study (Author et al., year) | Overall risk of bias (diagnostic) | Spectrum / applicability concerns | Reproducibility risk (code / data) | Overall AI bias | Certainty – Sensitivity (GRADE) | Certainty – Specificity (GRADE) |
| --- | --- | --- | --- | --- | --- | --- |
| Fu et al., 2023 | Moderate | Low–Moderate (surgical candidates only) | High (no code or data shared) | Moderate | Moderate (⊕⊕⊕○) | Moderate (⊕⊕⊕○) |
| Xu et al., 2021 | Moderate–High | Moderate–High (strict criteria, small single-center sample) | Moderate (code yes, data no) | Moderate | Low–Moderate (⊕⊕○○) | Low–Moderate (⊕⊕○○) |
| Xia et al., 2020 | Moderate | Moderate (stage I only; multifocal nodules) | Moderate (code yes, data on request) | Moderate | Moderate (⊕⊕⊕○) | Moderate (⊕⊕⊕○) |
| Wang et al., 2021a | Moderate–High | Moderate–High (single center, multifocal, GGN ≤30 mm only) | High (no code; data on request only) | Moderate–High | Low–Moderate (⊕⊕○○) | Low–Moderate (⊕⊕○○) |
| Shen et al., 2021 | Some concerns (surgical-only, malignant-heavy) | High (surgical patients only, Chinese single-center) | Moderate–High (no open code/data) | High | Low–Moderate (⊕⊕○○) | Low–Moderate (⊕⊕○○) |
| Qi et al., 2024 | High | High (surgical pGGNs only, malignant enrichment) | High (no imaging data; limited transparency) | Moderate–High | Low–Moderate (⊕⊕○○) | Low–Moderate (⊕⊕○○) |
| Wang et al., 2021b | Low | High (surgical-only invasive nodules dominate) | High (no code/data, internal CV only) | High | Low–Moderate (⊕⊕○○) | Low–Moderate (⊕⊕○○) |
| Wang et al., 2021c | High / Unclear | High (GGNs only; high IAC prevalence) | High (no code/data) | High | Very low (⊕○○○) | Very low (⊕○○○) |
| Park et al., 2021 | Some concerns (retrospective surgical cohort) | High (only resected SSNs; surgical spectrum) | High (no code/data) | Moderate–High | Low–Moderate (⊕⊕○○) | Low–Moderate (⊕⊕○○) |
| Chae et al., 2014 | Low (index/ref/flow); unclear selection | Moderate (part-solid GGNs; restricted spectrum) | Moderate (no code/data; manual segmentation) | Moderate | Very low (⊕○○○) | Very low (⊕○○○) |
| Xing Xue et al., 2018 | High / Unclear | High (resected GGNs only; 5–30 mm; 5 mm slices) | High (no code/data; partial methods) | High | Very low (⊕○○○) | Very low (⊕○○○) |
| Weng et al., 2019 | High / Unclear | High (surgical; MIA/IA only) | High (no code/data; proprietary tools) | High | Low (⊕⊕○○) | Low (⊕⊕○○) |
| Ye et al., 2020 | High | High (surgical-only adenocarcinoma; no proper split) | High (no code/data) | High | Low (⊕⊕○○) | Low (⊕⊕○○) |
| Cai et al., 2020 | High | High (female-heavy, surgical-only cohort) | High (manual ROI; no code/data) | High | Low (⊕⊕○○) | Low (⊕⊕○○) |
| Yanagawa et al., 2019 | High / Unclear (retrospective, limited reporting) | High (likely resected / surgical-leaning spectrum) | High (no code/data reported) | High | Low (⊕⊕○○) | Low (⊕⊕○○) |
| Chen et al., 2021 | High | High (surgical SSPNs only) | High (no code/data) | High | Very low (⊕○○○) | Very low (⊕○○○) |
| Wang et al., 2025 | Some concerns (retrospective, surgery-only LUAD) | High (stage I resected LUAD only) | High (no code/data; TRIPOD-AI NR) | Moderate–High | Low–Very low (⊕⊕/⊕○○○) | Low–Very low (⊕⊕/⊕○○○) |
| Sun et al., 2025 | High | High (single-center surgical GGNs; ≤3 cm, ≤50% solid) | High (no code/data) | High | Very low (⊕○○○) | Very low (⊕○○○) |
| Zheng et al., 2023 | High | High (surgical-only; ≤3 cm GGNs; excludes solid lesions) | High (no code/data) | High | Very low (⊕○○○) | Very low (⊕○○○) |
| Zhang et al., 2024 | High | High (only resected GGNs; small sample) | High (no code/data; prototype tools) | High | Very low (⊕○○○) | Very low (⊕○○○) |
| Chen et al., 2025 | High | High (surgical-only cohort) | High (no code/data; partial TRIPOD only) | High | Very low (⊕○○○) | Very low (⊕○○○) |
| Zhou et al., 2023 | High / Unclear (retrospective AI radiomics) | High (likely surgical or enriched spectrum) | High (no code/data reported) | High | Low (⊕⊕○○) | Low (⊕⊕○○) |
