# Supplementary material for: Comparative diagnostic accuracy of radiomics, deep learning, and hybrid AI for invasiveness stratification of pulmonary ground-glass nodules: a systematic review and meta-analysis
Source: Front Oncol. 2026 Jul 24;16:1752652. doi: 10.3389/fonc.2026.1752652 (PMC13447058; doi:10.3389/fonc.2026.1752652)
Supplement: Supplementary file 2 [file Table2.docx]

### **PubMed Search Strategy (n= 789)**

(("Lung Neoplasms"[MeSH Terms] OR "Carcinoma, Non-Small-Cell Lung"[MeSH Terms]

OR lung neoplasm*[Title/Abstract] OR neoplasms, lung[Title/Abstract] OR neoplasms, pulmonary[Title/Abstract]

OR neoplasm, pulmonary[Title/Abstract] OR neoplasm, lung[Title/Abstract] OR pulmonary neoplasm*[Title/Abstract]

OR lung cancer*[Title/Abstract] OR cancer, lung[Title/Abstract] OR cancers, lung[Title/Abstract]

OR lung tumo*[Title/Abstract] OR lung carcinoma*[Title/Abstract] OR Pulmonary Neoplasm*[Title/Abstract]

OR pulmonary cancer*[Title/Abstract] OR pulmonary tumo*[Title/Abstract] OR pulmonary carcinoma*[Title/Abstract]

OR "cancer of the lung"[Title/Abstract] OR "cancer of lung"[Title/Abstract] OR cancer, pulmonary[Title/Abstract]

OR cancers, pulmonary[Title/Abstract]

OR "Adenocarcinoma, Bronchiolo-Alveolar"[MeSH Terms]

OR "Adenocarcinoma, Bronchiolo Alveolar"[Title/Abstract] OR "Adenocarcinomas, Bronchiolo-Alveolar"[Title/Abstract]

OR "Bronchiolo-Alveolar Adenocarcinoma"[Title/Abstract] OR "Bronchiolo-Alveolar Adenocarcinomas"[Title/Abstract]

OR "Adenocarcinoma, Alveolar"[Title/Abstract] OR "Adenocarcinomas, Alveolar"[Title/Abstract]

OR "Alveolar Adenocarcinoma"[Title/Abstract] OR "Alveolar Adenocarcinomas"[Title/Abstract]

OR "Carcinoma, Alveolar"[Title/Abstract] OR "Alveolar Carcinoma"[Title/Abstract] OR "Alveolar Carcinomas"[Title/Abstract]

OR "Carcinomas, Alveolar"[Title/Abstract] OR "Carcinoma, Bronchiolo-Alveolar"[Title/Abstract]

OR "Bronchiolo-Alveolar Carcinoma"[Title/Abstract] OR "BronchioloAlveolar Carcinomas"[Title/Abstract]

OR "Carcinoma, Bronchiolo Alveolar"[Title/Abstract] OR "Carcinomas, BronchioloAlveolar"[Title/Abstract]

OR "Alveolar Cell Carcinoma"[Title/Abstract] OR "Alveolar Cell Carcinomas"[Title/Abstract]

OR "Carcinoma, Alveolar Cell"[Title/Abstract] OR "Carcinomas, Alveolar Cell"[Title/Abstract]

OR "Carcinoma, Bronchiolar"[Title/Abstract] OR "Bronchiolar Carcinoma"[Title/Abstract]

OR "Bronchiolar Carcinomas"[Title/Abstract] OR "Carcinomas, Bronchiolar"[Title/Abstract]

OR "Carcinoma, Bronchioloalveolar"[Title/Abstract] OR "Bronchioloalveolar Carcinoma"[Title/Abstract]

OR "Bronchioloalveolar Carcinomas"[Title/Abstract] OR "Carcinomas, Bronchioloalveolar"[Title/Abstract]

OR "Adenocarcinoma of Lung"[MeSH Terms]

OR lung adenocarcinomas[Title/Abstract] OR lung adenocarcinoma[Title/Abstract]

OR adenocarcinoma lung[Title/Abstract] OR adenocarcinomas lung[Title/Abstract]

OR LUAD[Title/Abstract]

OR "Solitary Pulmonary Nodule"[MeSH Terms]

OR "Pulmonary Nodule, Solitary"[Title/Abstract] OR "Solitary Lung Nodule"[Title/Abstract]

OR "Lung Nodule, Solitary"[Title/Abstract] OR "Nodule, Solitary Lung"[Title/Abstract]

OR "Solitary Lung Nodules"[Title/Abstract] OR "Solitary Pulmonary Nodules"[Title/Abstract]

OR "Nodule, Solitary Pulmonary"[Title/Abstract] OR "Nodules, Solitary Pulmonary"[Title/Abstract]

OR "Pulmonary Nodules, Solitary"[Title/Abstract] OR "Pulmonary Coin Lesion"[Title/Abstract]

OR "Lesion, Pulmonary Coin"[Title/Abstract] OR "Coin Lesions, Pulmonary"[Title/Abstract]

OR "Pulmonary Coin Lesions"[Title/Abstract] OR "Coin Lesion, Pulmonary"[Title/Abstract]

OR ground-glass nodule*[Title/Abstract] OR solid nodule*[Title/Abstract]

OR subsolid nodule*[Title/Abstract] OR part-solid nodule*[Title/Abstract]

OR non-solid nodule*[Title/Abstract] OR groundglass opacities[Title/Abstract]

OR "ground-glass"[Title/Abstract] OR GGO[Title/Abstract] OR GGN[Title/Abstract]

OR SPN[Title/Abstract] OR pGGN[Title/Abstract] OR PSN[Title/Abstract]

OR SSN[Title/Abstract] OR mGGN[Title/Abstract] OR NSN[Title/Abstract]

OR "Multiple Pulmonary Nodules"[MeSH Terms]

OR "multiple pulmonary nodule"[Title/Abstract] OR "pulmonary nodule multiple"[Title/Abstract]

OR "pulmonary nodules multiple"[Title/Abstract]))

AND

(("Artificial Intelligence"[MeSH Terms] OR "Machine Learning"[MeSH Terms] OR "Deep Learning"[MeSH Terms]

OR artificial intelligence[Title/Abstract] OR machine learning model[Title/Abstract]

OR machine learning algorithms[Title/Abstract] OR algorithms[Title/Abstract]

OR machine learning[Title/Abstract] OR deep learning[Title/Abstract]

OR machine intelligence[Title/Abstract] OR computational approach[Title/Abstract]

OR computer intelligence[Title/Abstract] OR computer reasoning[Title/Abstract]

OR "Computer Vision System*"[Title/Abstract] OR automated-computer aided[Title/Abstract]

OR knowledge representation[Title/Abstract] OR knowledge acquisition[Title/Abstract]

OR expert system*[Title/Abstract] OR neural network*[Title/Abstract]

OR convolutional neural network[Title/Abstract] OR CNN[Title/Abstract]

OR artificial neuronal network[Title/Abstract] OR AI[Title/Abstract])

OR

(radiomic*[Title/Abstract] OR "texture analy*"[Title/Abstract] OR "image biomarker*"[Title/Abstract]

OR IBSI[Title/Abstract] OR GLCM[Title/Abstract] OR Haralick[Title/Abstract]

OR wavelet*[Title/Abstract] OR LASSO[Title/Abstract]

OR "random forest"[Title/Abstract] OR "support vector*"[Title/Abstract]

OR SVM[Title/Abstract] OR XGBoost[Title/Abstract] OR CatBoost[Title/Abstract]

OR segmentation[Title/Abstract] OR segment*[Title/Abstract] OR mask*[Title/Abstract]

OR contour*[Title/Abstract] OR delineat*[Title/Abstract]

OR "region of interest"[Title/Abstract] OR ROI[Title/Abstract] OR peritumor*[Title/Abstract]))

AND

("Tomography, X-Ray Computed"[MeSH Terms]

OR tomography scanners, x-ray computed[Title/Abstract]

OR computed tomography[Title/Abstract]

OR computer assisted tomography[Title/Abstract]

OR CT[Title/Abstract])

AND

(Invasive[Title/Abstract] OR invasiveness[Title/Abstract] OR risk[Title/Abstract]

OR Risk Stratification[Title/Abstract] OR Risk assessment[Title/Abstract]

OR risk management[Title/Abstract] OR subtype classification[Title/Abstract]

OR histological assessment[Title/Abstract]))

### Strategy for Cochrane Library (n=106):

#1 MeSH descriptor: [Lung Neoplasms] explode all trees

#2 MeSH descriptor: [Adenocarcinoma of Lung] explode all trees

#3 MeSH descriptor: [Solitary Pulmonary Nodule] explode all trees

#4 MeSH descriptor: [Adenocarcinoma, Bronchiolo-Alveolar] explode all trees

#5 MeSH descriptor: [Carcinoma, Non-Small-Cell Lung] explode all trees

#6 MeSH descriptor: [Carcinoma, Bronchogenic] explode all trees

#7 MeSH descriptor: [Bronchial Neoplasms] explode all trees

#8 MeSH descriptor: [Multiple Pulmonary Nodules] explode all trees

#9 (Bronchiolo-Alveolar Carcinomas):ti,ab,kw OR (Adenocarcinomas, Lung):ti,ab,kw OR (Carcinoma, Alveolar):ti,ab,kw

OR (Bronchiolo-Alveolar Carcinoma):ti,ab,kw OR (Carcinomas, Bronchial):ti,ab,kw OR (Carcinomas, Alveolar):ti,ab,kw

OR (Pulmonary Nodules, Multiple):ti,ab,kw OR (Non-Small-Cell Lung Carcinoma):ti,ab,kw OR (Cancer, Pulmonary):ti,ab,kw

OR (Bronchiolar Carcinomas):ti,ab,kw OR (Carcinomas, Bronchiolar):ti,ab,kw OR (Lung Carcinomas, NonSmall-Cell):ti,ab,kw

OR (Nonsmall Cell Lung Cancer):ti,ab,kw OR (Alveolar Carcinomas):ti,ab,kw OR (Neoplasm, Bronchial):ti,ab,kw

OR (Adenocarcinomas, Alveolar):ti,ab,kw OR (Carcinoma, Bronchiolo-Alveolar):ti,ab,kw OR (Carcinoma, Bronchiolar):ti,ab,kw

OR (Lung Cancers):ti,ab,kw OR (Alveolar Adenocarcinomas):ti,ab,kw OR (Alveolar Cell Carcinoma):ti,ab,kw

OR (Bronchiolo-Alveolar Adenocarcinoma):ti,ab,kw OR (Cancer, Lung):ti,ab,kw OR (Carcinomas, Bronchogenic):ti,ab,kw

OR (Bronchiolar Carcinoma):ti,ab,kw OR (Neoplasms, Pulmonary):ti,ab,kw OR (Non-Small Cell Lung Cancer):ti,ab,kw

OR (Carcinoma, Bronchioloalveolar):ti,ab,kw OR (Carcinoma, Non-Small Cell Lung):ti,ab,kw OR (Alveolar Adenocarcinoma):ti,ab,kw

OR (Adenocarcinoma, Alveolar):ti,ab,kw OR (Bronchial Carcinoma):ti,ab,kw OR (Pulmonary Nodule, Multiple):ti,ab,kw

OR (Carcinoma, Bronchial):ti,ab,kw OR (Carcinomas, Non-Small-Cell Lung):ti,ab,kw OR (Carcinomas, Bronchiolo-Alveolar):ti,ab,kw

OR (Neoplasm, Pulmonary):ti,ab,kw OR (Non-SmallCell Lung Carcinomas):ti,ab,kw OR (Neoplasms, Bronchial):ti,ab,kw

OR (Bronchioloalveolar Carcinoma):ti,ab,kw OR (Bronchogenic Carcinomas):ti,ab,kw OR (Carcinoma, Non Small Cell Lung):ti,ab,kw

OR (Alveolar Cell Carcinomas):ti,ab,kw OR (Lung Neoplasm):ti,ab,kw OR (Cancers, Pulmonary):ti,ab,kw

OR (Bronchogenic Carcinoma):ti,ab,kw OR (Pulmonary Cancers):ti,ab,kw OR (Adenocarcinoma, Bronchiolo Alveolar):ti,ab,kw

OR (Lung Adenocarcinoma):ti,ab,kw OR (Non Small Cell Lung Carcinoma):ti,ab,kw OR (Multiple Pulmonary Nodule):ti,ab,kw

OR (Lung Cancer):ti,ab,kw OR (Cancer of Lung):ti,ab,kw OR (Lung Adenocarcinomas):ti,ab,kw OR (Neoplasm, Lung):ti,ab,kw

OR (Adenocarcinomas, Bronchiolo-Alveolar):ti,ab,kw OR (Bronchial Neoplasm):ti,ab,kw OR (Adenocarcinoma, Lung):ti,ab,kw

OR (Pulmonary Neoplasms):ti,ab,kw OR (Carcinomas, Bronchioloalveolar):ti,ab,kw OR (Bronchiolo-Alveolar Adenocarcinomas):ti,ab,kw

OR (Lung Carcinoma, Non-Small-Cell):ti,ab,kw OR (Carcinoma, Bronchiolo Alveolar):ti,ab,kw OR (Cancer of the Lung):ti,ab,kw

OR (Pulmonary Cancer):ti,ab,kw OR (Neoplasms, Lung):ti,ab,kw OR (Pulmonary Neoplasm):ti,ab,kw OR (Alveolar Carcinoma):ti,ab,kw

OR (Carcinomas, Alveolar Cell):ti,ab,kw OR (Cancers, Lung):ti,ab,kw OR (Bronchial Carcinomas):ti,ab,kw

OR (Bronchioloalveolar Carcinomas):ti,ab,kw OR (Carcinoma, Alveolar Cell):ti,ab,kw

OR (pulmonary nodule):ti,ab,kw OR (lung nodule):ti,ab,kw OR (ground-glass nodule):ti,ab,kw

OR (solid nodule):ti,ab,kw OR (subsolid nodule):ti,ab,kw OR (GGO):ti,ab,kw OR (pGGN):ti,ab,kw OR (PSN):ti,ab,kw

OR (GGN):ti,ab,kw OR (mGGN):ti,ab,kw OR (SSN):ti,ab,kw OR (SPN):ti,ab,kw OR (NSN):ti,ab,kw OR (LUAD):ti,ab,kw)

#10 #1 OR #2 OR #3 OR #4 OR #5 OR #6 OR #7 OR #8 OR #9

#11 MeSH descriptor: [Artificial Intelligence] explode all trees

#12 MeSH descriptor: [Neural Networks, Computer] explode all trees

#13 MeSH descriptor: [Knowledge Bases] explode all trees

#14 MeSH descriptor: [Biological Ontologies] explode all trees

#15 MeSH descriptor: [Robotics] explode all trees

#16 MeSH descriptor: [Unsupervised Machine Learning] explode all trees

#17 MeSH descriptor: [Fuzzy Logic] explode all trees

#18 MeSH descriptor: [Natural Language Processing] explode all trees

#19 MeSH descriptor: [Machine Learning] explode all trees

#20 MeSH descriptor: [Deep Learning] explode all trees

#21 MeSH descriptor: [Expert Systems] explode all trees

#22 MeSH descriptor: [Supervised Machine Learning] explode all trees

#23 MeSH descriptor: [Computer Heuristics] explode all trees

#24 (Vision System, Computer):ti,ab,kw OR (Machine Learning, Unsupervised):ti,ab,kw OR (Semi supervised Learning):ti,ab,kw

OR (Computer Vision Systems):ti,ab,kw OR (Knowledge Representations):ti,ab,kw OR (Machine Learning, Supervised):ti,ab,kw

OR (Remote Operation):ti,ab,kw OR (Systems, Computer Vision):ti,ab,kw OR (Network Models, Neural):ti,ab,kw

OR (Intelligence, Artificial):ti,ab,kw OR (Computational Neural Network):ti,ab,kw OR (Network, Computer Neural):ti,ab,kw

OR (Networks, Computational Neural):ti,ab,kw OR (Neural Network Models):ti,ab,kw OR (Computer Vision System):ti,ab,kw

OR (System, Computer Vision):ti,ab,kw OR (Models, Neural Network):ti,ab,kw OR (Neural Network Model):ti,ab,kw

OR (Networks, Computer Neural):ti,ab,kw OR (Learning, Deep):ti,ab,kw OR (Learning, Machine):ti,ab,kw OR (CNN):ti,ab,kw OR (AI):ti,ab,kw

#24b (radiomic*):ti,ab,kw OR ("texture analy*"):ti,ab,kw OR ("image biomarker*"):ti,ab,kw OR IBSI:ti,ab,kw OR GLCM:ti,ab,kw

OR Haralick:ti,ab,kw OR wavelet*:ti,ab,kw OR LASSO:ti,ab,kw OR "random forest":ti,ab,kw OR "support vector*":ti,ab,kw

OR SVM:ti,ab,kw OR XGBoost:ti,ab,kw OR CatBoost:ti,ab,kw OR segmentation:ti,ab,kw OR segment*:ti,ab,kw

OR mask*:ti,ab,kw OR contour*:ti,ab,kw OR delineat*:ti,ab,kw OR "region of interest":ti,ab,kw OR ROI:ti,ab,kw

OR peritumor*:ti,ab,kw

#25 #11 OR #12 OR #13 OR #14 OR #15 OR #16 OR #17 OR #18 OR #19 OR #20 OR #21 OR #22 OR #23 OR #24 OR #24b

#26 MeSH descriptor: [Tomography, X-Ray Computed] explode all trees

#27 (tomography scanners, x-ray computed):ti,ab,kw OR (computed tomography):ti,ab,kw

OR (computer assisted tomography):ti,ab,kw OR (CT):ti,ab,kw OR (CAT):ti,ab,kw

#28 #26 OR #27

#29 ("invasiveness analysis"):ti,ab,kw OR ("Invasive"):ti,ab,kw OR ("invasiveness"):ti,ab,kw OR ("risk"):ti,ab,kw

OR ("Risk Stratification"):ti,ab,kw OR ("Risk assessment"):ti,ab,kw OR ("risk management"):ti,ab,kw

OR ("subtype classification"):ti,ab,kw OR ("histological assessment"):ti,ab,kw

#30 #10 AND #25 AND #28 AND #29

### Embase Search Strategy (n=1,409):

(exp = explode Emtree terms; :ti,ab,kw = search in title, abstract, keyword fields)

#1 'emergency nurse'/exp OR 'emergency nursing'/exp OR 'nurse'/exp OR (emergency nurse*:ti,ab,kw OR nursing staff:ti,ab,kw)

#2 'training'/exp OR 'education'/exp OR 'simulation training'/exp OR 'clinical training'/exp OR (training:ti,ab,kw OR education:ti,ab,kw OR simulation:ti,ab,kw OR "real rescue":ti,ab,kw OR "rescue training":ti,ab,kw)

#3 'resuscitation'/exp OR 'emergency care'/exp OR 'emergency treatment'/exp OR (resuscitation:ti,ab,kw OR "emergency response":ti,ab,kw OR "real emergency":ti,ab,kw)

#4 'teamwork'/exp OR 'team collaboration'/exp OR 'team climate'/exp OR (teamwork:ti,ab,kw OR collaboration:ti,ab,kw OR "team climate":ti,ab,kw)

#5 'stress'/exp OR 'psychological resilience'/exp OR 'burnout'/exp OR (stress resistance:ti,ab,kw OR resilience:ti,ab,kw OR burnout:ti,ab,kw OR "occupational burnout":ti,ab,kw)

#6 'critical thinking'/exp OR (critical thinking:ti,ab,kw OR cognition:ti,ab,kw OR reasoning:ti,ab,kw)

#7 #1 AND #2 AND #3 AND (#4 OR #5 OR #6)

### Web of Science Search Strategy

(TS = Topic, searches title/abstract/keywords)

#1 TS=(“emergency nurse*” OR “nursing staff” OR “emergency nursing”)

#2 TS=(training OR education OR “simulation training” OR “real rescue” OR “rescue training” OR “clinical training”)

#3 TS=(resuscitation OR “emergency care” OR “emergency response” OR “real emergency”)

#4 TS=(teamwork OR “team collaboration” OR “team climate”)

#5 TS=(stress resistance OR resilience OR burnout OR “occupational burnout”)

#6 TS=(critical thinking OR cognition OR reasoning)

#7 #1 AND #2 AND #3 AND (#4 OR #5 OR #6)
