# Supplementary material for: Comparative diagnostic accuracy of radiomics, deep learning, and hybrid AI for invasiveness stratification of pulmonary ground-glass nodules: a systematic review and meta-analysis
Source: Front Oncol. 2026 Jul 24;16:1752652. doi: 10.3389/fonc.2026.1752652 (PMC13447058; doi:10.3389/fonc.2026.1752652)
Supplement: Supplementary file 3 [file Table3.docx]

# Supplementary Tables

## Supplementary Table S2. Study-level risk of bias and applicability (adapted QUADAS-2)

| Study ID | Patient selection (RoB) | Patient selection (Applicability) | Index test (RoB) | Index test (Applicability) | Reference standard (RoB) | Reference standard (Applicability) | Flow & timing (RoB) | Flow & timing (Applicability) |
| --- | --- | --- | --- | --- | --- | --- | --- | --- |
| Fu 2023 | High (retrospective, possible non-consecutive sampling) | Low (screen-detected pulmonary nodules similar to review question) | Unclear (AI thresholding / model training not fully reported) | Low (index test targets same construct: invasiveness of lung nodules) | Unclear (reference standard definition/reporting insufficient) | Low (histopathology / multidisciplinary diagnosis appropriate) | High (single-centre, no external validation reported) | Low (clinical pathway comparable to review target setting) |
| Xu 2021 | High (retrospective, possible non-consecutive sampling) | Low (screen-detected pulmonary nodules similar to review question) | Unclear (AI thresholding / model training not fully reported) | Low (index test targets same construct: invasiveness of lung nodules) | Unclear (reference standard definition/reporting insufficient) | Low (histopathology / multidisciplinary diagnosis appropriate) | High (single-centre, no external validation reported) | Low (clinical pathway comparable to review target setting) |
| Xia 2020 | High (retrospective, possible non-consecutive sampling) | Low (screen-detected pulmonary nodules similar to review question) | Unclear (AI thresholding / model training not fully reported) | Low (index test targets same construct: invasiveness of lung nodules) | Unclear (reference standard definition/reporting insufficient) | Low (histopathology / multidisciplinary diagnosis appropriate) | High (single-centre, no external validation reported) | Low (clinical pathway comparable to review target setting) |
| Wang 2021 | High (retrospective, possible non-consecutive sampling) | Low (screen-detected pulmonary nodules similar to review question) | Unclear (AI thresholding / model training not fully reported) | Low (index test targets same construct: invasiveness of lung nodules) | Unclear (reference standard definition/reporting insufficient) | Low (histopathology / multidisciplinary diagnosis appropriate) | High (single-centre, no external validation reported) | Low (clinical pathway comparable to review target setting) |
| Shen 2021 | High (retrospective, possible non-consecutive sampling) | Low (screen-detected pulmonary nodules similar to review question) | Unclear (AI thresholding / model training not fully reported) | Low (index test targets same construct: invasiveness of lung nodules) | Unclear (reference standard definition/reporting insufficient) | Low (histopathology / multidisciplinary diagnosis appropriate) | Low (includes external validation cohort) | Low (clinical pathway comparable to review target setting) |
| Qi 2024 | High (retrospective, possible non-consecutive sampling) | Low (screen-detected pulmonary nodules similar to review question) | Unclear (AI thresholding / model training not fully reported) | Low (index test targets same construct: invasiveness of lung nodules) | Unclear (reference standard definition/reporting insufficient) | Low (histopathology / multidisciplinary diagnosis appropriate) | High (single-centre, no external validation reported) | Low (clinical pathway comparable to review target setting) |
| Wang 2021 (IMAL-Net) | High (retrospective, possible non-consecutive sampling) | Low (screen-detected pulmonary nodules similar to review question) | Unclear (AI thresholding / model training not fully reported) | Low (index test targets same construct: invasiveness of lung nodules) | Unclear (reference standard definition/reporting insufficient) | Low (histopathology / multidisciplinary diagnosis appropriate) | Low (includes external validation cohort) | Low (clinical pathway comparable to review target setting) |
| Wang et al. 2021 | High (retrospective, possible non-consecutive sampling) | Low (screen-detected pulmonary nodules similar to review question) | Unclear (AI thresholding / model training not fully reported) | Low (index test targets same construct: invasiveness of lung nodules) | Unclear (reference standard definition/reporting insufficient) | Low (histopathology / multidisciplinary diagnosis appropriate) | High (single-centre, no external validation reported) | Low (clinical pathway comparable to review target setting) |
| Park 2021 | High (retrospective, possible non-consecutive sampling) | Low (screen-detected pulmonary nodules similar to review question) | Unclear (AI thresholding / model training not fully reported) | Low (index test targets same construct: invasiveness of lung nodules) | Unclear (reference standard definition/reporting insufficient) | Low (histopathology / multidisciplinary diagnosis appropriate) | Low (includes external validation cohort) | Low (clinical pathway comparable to review target setting) |
| Chae 2014 | High (retrospective, possible non-consecutive sampling) | Low (screen-detected pulmonary nodules similar to review question) | Unclear (AI thresholding / model training not fully reported) | Low (index test targets same construct: invasiveness of lung nodules) | Unclear (reference standard definition/reporting insufficient) | Low (histopathology / multidisciplinary diagnosis appropriate) | High (single-centre, no external validation reported) | Low (clinical pathway comparable to review target setting) |
| Xing Xue 2018 | High (retrospective, possible non-consecutive sampling) | Low (screen-detected pulmonary nodules similar to review question) | Unclear (AI thresholding / model training not fully reported) | Low (index test targets same construct: invasiveness of lung nodules) | Unclear (reference standard definition/reporting insufficient) | Low (histopathology / multidisciplinary diagnosis appropriate) | High (single-centre, no external validation reported) | Low (clinical pathway comparable to review target setting) |
| Weng 2019 | High (retrospective, possible non-consecutive sampling) | Low (screen-detected pulmonary nodules similar to review question) | Unclear (AI thresholding / model training not fully reported) | Low (index test targets same construct: invasiveness of lung nodules) | Unclear (reference standard definition/reporting insufficient) | Low (histopathology / multidisciplinary diagnosis appropriate) | High (single-centre, no external validation reported) | Low (clinical pathway comparable to review target setting) |
| Zhou 2019 | High (retrospective, possible non-consecutive sampling) | Low (screen-detected pulmonary nodules similar to review question) | Unclear (AI thresholding / model training not fully reported) | Low (index test targets same construct: invasiveness of lung nodules) | Unclear (reference standard definition/reporting insufficient) | Low (histopathology / multidisciplinary diagnosis appropriate) | High (single-centre, no external validation reported) | Low (clinical pathway comparable to review target setting) |
| Cai 2020 | High (retrospective, possible non-consecutive sampling) | Low (screen-detected pulmonary nodules similar to review question) | Unclear (AI thresholding / model training not fully reported) | Low (index test targets same construct: invasiveness of lung nodules) | Unclear (reference standard definition/reporting insufficient) | Low (histopathology / multidisciplinary diagnosis appropriate) | High (single-centre, no external validation reported) | Low (clinical pathway comparable to review target setting) |
| Yanagawa 2019 | High (retrospective, possible non-consecutive sampling) | Low (screen-detected pulmonary nodules similar to review question) | Unclear (AI thresholding / model training not fully reported) | Low (index test targets same construct: invasiveness of lung nodules) | Unclear (reference standard definition/reporting insufficient) | Low (histopathology / multidisciplinary diagnosis appropriate) | High (single-centre, no external validation reported) | Low (clinical pathway comparable to review target setting) |
| Chen 2021 | High (retrospective, possible non-consecutive sampling) | Low (screen-detected pulmonary nodules similar to review question) | Unclear (AI thresholding / model training not fully reported) | Low (index test targets same construct: invasiveness of lung nodules) | Unclear (reference standard definition/reporting insufficient) | Low (histopathology / multidisciplinary diagnosis appropriate) | High (single-centre, no external validation reported) | Low (clinical pathway comparable to review target setting) |
| Wang 2025 | High (retrospective, possible non-consecutive sampling) | Low (screen-detected pulmonary nodules similar to review question) | Unclear (AI thresholding / model training not fully reported) | Low (index test targets same construct: invasiveness of lung nodules) | Unclear (reference standard definition/reporting insufficient) | Low (histopathology / multidisciplinary diagnosis appropriate) | Low (includes external validation cohort) | Low (clinical pathway comparable to review target setting) |
| Sun 2025 | High (retrospective, possible non-consecutive sampling) | Low (screen-detected pulmonary nodules similar to review question) | Unclear (AI thresholding / model training not fully reported) | Low (index test targets same construct: invasiveness of lung nodules) | Unclear (reference standard definition/reporting insufficient) | Low (histopathology / multidisciplinary diagnosis appropriate) | High (single-centre, no external validation reported) | Low (clinical pathway comparable to review target setting) |
| Zheng 2023 | High (retrospective, possible non-consecutive sampling) | Low (screen-detected pulmonary nodules similar to review question) | Unclear (AI thresholding / model training not fully reported) | Low (index test targets same construct: invasiveness of lung nodules) | Unclear (reference standard definition/reporting insufficient) | Low (histopathology / multidisciplinary diagnosis appropriate) | High (single-centre, no external validation reported) | Low (clinical pathway comparable to review target setting) |
| Zhang 2024 | High (retrospective, possible non-consecutive sampling) | Low (screen-detected pulmonary nodules similar to review question) | Unclear (AI thresholding / model training not fully reported) | Low (index test targets same construct: invasiveness of lung nodules) | Unclear (reference standard definition/reporting insufficient) | Low (histopathology / multidisciplinary diagnosis appropriate) | High (single-centre, no external validation reported) | Low (clinical pathway comparable to review target setting) |
| Chen 2025 | High (retrospective, possible non-consecutive sampling) | Low (screen-detected pulmonary nodules similar to review question) | Unclear (AI thresholding / model training not fully reported) | Low (index test targets same construct: invasiveness of lung nodules) | Unclear (reference standard definition/reporting insufficient) | Low (histopathology / multidisciplinary diagnosis appropriate) | High (single-centre, no external validation reported) | Low (clinical pathway comparable to review target setting) |
| Zhou 2023 | High (retrospective, possible non-consecutive sampling) | Low (screen-detected pulmonary nodules similar to review question) | Unclear (AI thresholding / model training not fully reported) | Low (index test targets same construct: invasiveness of lung nodules) | Unclear (reference standard definition/reporting insufficient) | Low (histopathology / multidisciplinary diagnosis appropriate) | Low (includes external validation cohort) | Low (clinical pathway comparable to review target setting) |

RoB = risk of bias. Judgments are inferred from reported study design and may need refinement against the full QUADAS-2 signaling questions.

## Supplementary Table S3. GRADE evidence profile for AI methodologies

| AI methodology | Outcome | Pooled estimate (95% CI) | Risk of bias | Inconsistency | Indirectness | Imprecision | Publication bias | Overall certainty |
| --- | --- | --- | --- | --- | --- | --- | --- | --- |
| Radiomics | Sensitivity | 0.83 (0.79–0.87) | Serious | Serious (I²>60%) | Not serious | Not serious | Suspected (Deeks p=0.03) | Low |
| Radiomics | Specificity | 0.80 (0.76–0.84) | Serious | Serious | Not serious | Not serious | Suspected | Low |
| Radiomics | AUC | 0.89 (0.86–0.91) | Serious | Serious | Not serious | Not serious | Suspected | Low |
| Deep learning | Sensitivity | 0.88 (0.85–0.91) | Serious | Serious | Not serious | Not serious | Suspected | Low |
| Deep learning | Specificity | 0.85 (0.81–0.88) | Serious | Serious | Not serious | Not serious | Suspected | Low |
| Deep learning | AUC | 0.92 (0.90–0.94) | Serious | Serious | Not serious | Not serious | Suspected | Low |
| Hybrid | Sensitivity | 0.91 (0.88–0.93) | Serious | Serious (heterogeneity, small-study effects) | Not serious | Not serious | Suspected | Low to moderate |
| Hybrid | Specificity | 0.87 (0.83–0.90) | Serious | Serious | Not serious | Not serious | Suspected | Low to moderate |
| Hybrid | AUC | 0.94 (0.92–0.96) | Serious | Serious | Not serious | Not serious | Suspected | Low to moderate |

Certainty ratings reflect predominantly retrospective designs, high heterogeneity, and potential publication bias, consistent with the manuscript text.

## Supplementary Table S4. Positive and negative predictive values across prevalence scenarios (using pooled sensitivity and specificity)

| AI methodology | Assumed prevalence of invasive cancer | Sensitivity | Specificity | PPV | NPV |
| --- | --- | --- | --- | --- | --- |
| Radiomics | 10% | 0.83 | 0.80 | 0.32 | 0.98 |
| Radiomics | 20% | 0.83 | 0.80 | 0.51 | 0.95 |
| Radiomics | 30% | 0.83 | 0.80 | 0.64 | 0.92 |
| Deep Learning | 10% | 0.88 | 0.85 | 0.39 | 0.98 |
| Deep Learning | 20% | 0.88 | 0.85 | 0.59 | 0.97 |
| Deep Learning | 30% | 0.88 | 0.85 | 0.72 | 0.94 |
| Hybrid | 10% | 0.91 | 0.87 | 0.44 | 0.99 |
| Hybrid | 20% | 0.91 | 0.87 | 0.64 | 0.97 |
| Hybrid | 30% | 0.91 | 0.87 | 0.75 | 0.96 |

PPV and NPV were calculated from pooled sensitivity and specificity estimates for radiomics, deep learning, and hybrid approaches at assumed prevalences of 10%, 20%, and 30%.
